# Fluorescence lifetime imaging microscopy: fundamentals and advances in instrumentation, analysis, and applications

**DOI:** 10.1117/1.JBO.25.7.071203

**Published:** 2020-05-13

**Authors:** Rupsa Datta, Tiffany M. Heaster, Joe T. Sharick, Amani A. Gillette, Melissa C. Skala

**Affiliations:** a Morgridge Institute for Research, Madison, Wisconsin, United States; b University of Wisconsin, Department of Biomedical Engineering, Madison, Wisconsin, United States

**Keywords:** fluorescence lifetime, microscopy, image analysis, cell heterogeneity, review

## Abstract

**Significance:** Fluorescence lifetime imaging microscopy (FLIM) is a powerful technique to distinguish the unique molecular environment of fluorophores. FLIM measures the time a fluorophore remains in an excited state before emitting a photon, and detects molecular variations of fluorophores that are not apparent with spectral techniques alone. FLIM is sensitive to multiple biomedical processes including disease progression and drug efficacy.

**Aim:** We provide an overview of FLIM principles, instrumentation, and analysis while highlighting the latest developments and biological applications.

**Approach:** This review covers FLIM principles and theory, including advantages over intensity-based fluorescence measurements. Fundamentals of FLIM instrumentation in time- and frequency-domains are summarized, along with recent developments. Image segmentation and analysis strategies that quantify spatial and molecular features of cellular heterogeneity are reviewed. Finally, representative applications are provided including high-resolution FLIM of cell- and organelle-level molecular changes, use of exogenous and endogenous fluorophores, and imaging protein-protein interactions with Förster resonance energy transfer (FRET). Advantages and limitations of FLIM are also discussed.

**Conclusions:** FLIM is advantageous for probing molecular environments of fluorophores to inform on fluorophore behavior that cannot be elucidated with intensity measurements alone. Development of FLIM technologies, analysis, and applications will further advance biological research and clinical assessments.

## 1 Introduction

Fluorescence microscopy is a core biomedical imaging tool that provides high-resolution images of molecular contrast in living samples. Stokes coined the term “fluorescence” in 1852 for the “remarkable phenomena of light” observed in the materials that emitted light at a different color than the absorbed light.^1^ At that time, refraction (or refrangibility), internal dispersion, circular dichroism, and other phenomena of light were well studied. Stokes examined flower petals, leaves, turmeric, calcium fluoride, and many other compounds. In the 20th century, fluorescence was redefined as a short-lived emission of photons caused by the incidence of higher energy photons and became a popular tool for studying molecular dynamics and characterizing compounds.

In the mid-20th century, Weber used fluorescence properties of molecules such as depolarization along with absorption and emission spectra to pinpoint molecular dynamics and reveal kinetic parameters for biologically relevant processes such as enzyme binding.^2^ These experiments advanced fluorescence as a major means for biophysical and biochemical investigation. By the late 20th century, numerous brightly fluorescent small molecules had been categorized and repurposed as markers bound to other molecules. Fluorescence-based targeting provided unique molecular specificity in high-resolution microscopy. For example, mitochondria were identified using a small fluorescent molecule called tetramethyl-rhodamine-ethyl-ester (TMRE), which binds only to the mitochondrial membrane. This discovery has greatly advanced the study of energy distribution in biological systems. A weaker endogenous source of fluorescence was also identified within mitochondria. This inherent ability of many biological systems to fluoresce without the addition of external fluorophores was termed “autofluorescence.”

Along with reduced pyridine nucleotides, oxidized flavins, and other metabolic agents, proteins containing an abundance of amino acids, such as tryptophan, phenylalanine, and tyrosine, are the major endogenous fluorophores in biological systems. Studies in the 1980s identified the presence of a fluorescent protein expressed by jellyfish. This small protein was cloned into a functionally expressible green fluorescent protein (GFP)^3^ and was genetically expressed in *Escherichia coli* (*E. coli*) to create bacteria capable of green fluorescence.^4^ Thousands of varieties of this protein that fluoresce at different parts of the spectrum have been engineered, and their fluorescence behavior has been modified to cater to unique probing interests. The *in vivo* imaging capabilities of GFP-tagged proteins within organisms have bolstered fluorescence imaging as a robust and flexible assessment method for biomedical research.

Fluorescence lifetime imaging microscopy (FLIM), which exploits the lifetime property of fluorescence, is a microscopy technique that has gained popularity because of its high sensitivity to the molecular environment and changes in molecular conformation. FLIM has been extensively used in autofluorescent molecular imaging to study cellular metabolism. FLIM of autofluorescent molecules provides unique insights into cellular health in a nondestructive manner and is often used to study live animals and as a contrast mechanism for fluorescence-guided surgery.^5^^–^^12^ Exogenous fluorescent molecules that are capable of monitoring microenvironmental parameters, such as temperature, viscosity, pH, and ion concentration, are categorized as FLIM-based sensors.^13^^–^^15^ Protein–protein interactions can be monitored using Förster resonance energy transfer (FRET) sensors that are specific for cellular signaling, cellular proliferation, cytokinesis, and other molecular interactions.^16^^–^^19^ Thus, leveraging both endogenous and exogenous fluorophores, FLIM can monitor numerous processes in cells and tissues, including disease progression and drug efficacy.

In this review, we discuss the principles and theory behind FLIM and its unique advantages over intensity-based fluorescence microscopy methods. Then, we review FLIM instrumentation and FLIM analysis methods including segmentation and population density modeling of cell heterogeneity, and we close with a summary of FLIM applications *in vivo* and *in vitro*.

### 1.1 Fluorescence Lifetime

**Fig. 1:**
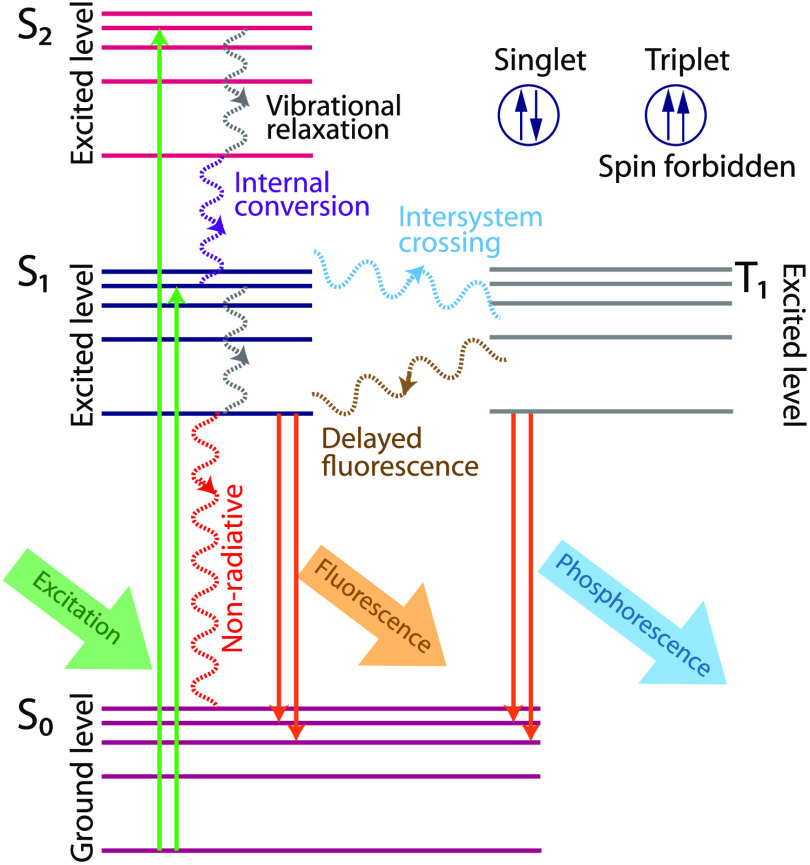
Schematic of Jablonski diagram.

When a molecule in ground state (denoted as $S_{0}$ in Fig. 1) absorbs light of energy equal or greater than the higher energy levels ($S_{1},S_{2},\ldots ,S_{n}$), an electron is excited to a higher energy level for a short period. The electron will undergo vibrational relaxation to the lowest vibrational level of the excited state (denoted as $S_{1}$) by a nonradiative process called internal conversion. From the $S_{1}$ electronic state, molecules return to the ground state either by a radiative or nonradiative process. Figure 1 represents the different luminescence phenomena that occur in these levels.

Fluorescence is a radiative process in which molecules (fluorophores) decay to the ground state by emitting detectable photons (on the timescale of $10^{-9}\;s$). The fluorescence emission happens from the lowest excited electronic level ($S_{1}$). This mandatory emission from the lowest excited electronic level ensures that the emission spectrum remains the same and is independent of the excitation wavelength. The energy of the emitted fluorescence photon is lower (i.e., emission occurs at a longer wavelength than the excitation) due to energy loss in vibrational relaxation and internal conversions. This shift in emission wavelength is referred to as the Stokes shift. Another predominant luminescence process, phosphorescence, occurs when the excited electron energy transitions into a triplet energy level ($T_{1},T_{2},\ldots ,T_{n}$) by a process known as intersystem crossing (ISC). Electrons in the triplet state have parallel spins, and these electron transitions are “spin-forbidden,” resulting in a slow transition to ground level by emission of a phosphorescence photon or reversal of the ISC and emission of a delayed fluorescence photon. Phosphorescence occurs on timescales on the order of milliseconds to hundreds of seconds. The Jablonski diagram shown in Fig. 1 concisely illustrates these processes.

The quantum yield of the molecule is defined as the ratio of emitted photons to the absorbed photons. Quantum yields for common fluorescent compounds include 80% for fluorescein,^20^ 60% for eGFP,^21^ 6% for tryptophan,^22^ and 2% for reduced nicotinamide adenine dinucleotide (NADH).^23^ This emission efficiency of a molecule depends on (1) its spatial orientation with respect to the incident electromagnetic wave’s electric field orientation (polarization), (2) the electronic energy levels available for absorbing the incoming photon energy (absorption spectrum), (3) the efficiency of rearrangement of vibrational levels (fluorescence lifetime), (4) relaxation back to the ground state electronic energy level (Stokes shift), and (5) the population of vibrational levels within this ground state (emission spectrum). Fluorophores are characterized by their absorption spectrum, fluorescence lifetime, Stokes shift, and emission spectrum.

Conventionally, we define fluorescence lifetime (τ) as the average time that a fluorophore remains in its excited state. In this interval, the intensity I(t) decreases to 1/e or 36.8% of its original value. The decaying intensity at time t is given by a first-order kinetics equation summed across all species, i, in the sample

$$I(t)=\underset{i}{\sum }\alpha_{i}e^{-t/\tau_{i}},$$ (1)

where α is the pre-exponential factor or the amplitude of the exponential function. The mean lifetime ($\tau_{m}$) of a multiexponential mixture of species is the sum of each species lifetime ($\tau_{i}$) weighted by fractional contribution of each species ($\alpha_{i}$)

$$\tau_{m}=\underset{i}{\sum }\tau_{i}\alpha_{i}.$$ (2)

In addition, the number of excited molecules at a time t is given as

$$n(t)=n(0)e^{-t/\tau },$$ (3)

where n(t) is the number of molecules in the excited state at time t.

Fluorescence lifetime can be measured in either the time-domain or frequency-domain, and these methods will be covered in detail in Secs. 2 and 3. Briefly, for time-domain methods, the sample is excited by a short excitation pulse and the decay is calculated either from time-of-arrival of photons that are binned into a histogram or by time-gated detection or pulse sampling techniques. If multiple fluorescent species are present, all species are summed into a single histogram. In frequency-domain methods, each photon is represented by its phase delay with respect to the excitation photon, which is similar to the arrival time histogram. For multiple species, this phase distribution is analyzed in Fourier space to extract the modulation and demodulation parameters that separate multiple species. Both time-domain and frequency-domain offer unique advantages and challenges in different FLIM scenarios including low photon budget imaging, high dynamic range imaging, or high time resolution imaging.

### 1.2 Autofluorescence FLIM Measurements

Biological systems are rich in endogenous fluorophores that are used for autofluorescence molecular imaging in a convenient, label-free manner. Endogenous fluorophores are powerful biomarkers because their emission properties are often influenced by their microenvironment, as well as the morphology, metabolic state, and pathological conditions of the sample. Notable endogenous fluorophores along with their excitation and emission wavelengths and fluorescence lifetimes are listed in Table 1. Imaging endogenous fluorophores is advantageous because it avoids the administration of external fluorescent dyes, thus circumventing complications introduced by these contrast agents including nonspecific binding, toxicity, and interference with the biochemical and physiological functions of the sample. Furthermore, autofluorescence imaging can be easily translated to *in vivo* monitoring in animal models and in humans for impactful clinical measurements.

**Table 1:** Spectral characteristics and lifetimes of endogenous fluorophores.

Endogenous fluorophore	Excitation (nm)	Emission (nm)	Lifetime (ns)	Reference
**Metabolic coenzymes**				
NAD(P)H free	340 (max)	470 (max)	0.4 (free), 1 to 5 (bound)	24–28
FAD, flavin	450 (max)	535 (max)	2.3 to 2.9 (free), <0.1 ns (bound)	26, 29, 30
Flavin mononucleotide (FMN)	444 (max)	558 (max)	4.27 to 4.67	31, 32
**Structural proteins**				
Collagen	280 to 350	370 to 440	0.2 to 0.4, 0.4 to 2.5	32, 33
Elastin	300 to 370	420 to 460	0.2 to 0.4, 0.4 to 2.5	32, 33
**Vitamins**				
Retinol	327 (max)	510 (max)	1.8, 5.0 (free), 0.7, 3.6, 12 (bound)	26, 34
Riboflavin	420 to 500	520 to 750	4.12	32
Vitamin B6	330 (max)	420 (max)	0.6 to 8.4	35, 36
Vitamin K	335 (max)	480 (max)	—	26
Vitamin D	390 (max)	480 (max)	—	26
Vitamin B12	275 (max)	305 (max)	—	26
**Pigments**				
Melanin	300 to 800	440, 520, 575	0.1 to 0.2, 0.5 to 1.8, 7.9	32–34
Eumelanin	355	520	0.058, 0.51, 2.9, 7	37, 38
Keratin	277 (max)	382 (max)	1.4	39, 40
Protoporhorphyrin IX	400 to 450	630, 690, 710	9.7 to 16	26, 41
Lipofuscin	340 to 395	540, 430 to 460	1.34	32, 35
Bilirubin	350 to 520	480 to 650	0.02 to 0.09, 1 to 2	42, 43
**Amino acids**				
Phenylalanine	258 (max)	280 (max)	7.5	32
Tryptophan	280 (max)	250 to 310	3.03	32
Tyrosine	275 (max)	300 (max)	2.5	32

#### 1.2.1 FLIM of NAD(P)H and FAD for metabolic imaging

Nicotinamide adenine dinucleotide (NAD) and flavin adenine dinucleotide (FAD) are two metabolic coenzymes that play a myriad of roles in cellular oxidation and reduction reactions. The reduced form NADH and oxidized form ${\mathrm{NAD}}^{+}$ are involved in mitochondrial function, energy metabolism, calcium homeostasis, gene expression, oxidative stress, aging, and apoptosis. The reduced NAD phosphate (NADPH) is associated with reductive fatty acid biosynthesis, steroid biosynthesis, oxidative stress, and antioxidation, while the oxidized form (${\mathrm{NADP}}^{+}$) is involved with calcium homeostasis.^9^ Real-time monitoring of cellular metabolism during pathophysiological changes is possible by measuring the redox ratio ($\mathrm{NADH}/{\mathrm{NAD}}^{+}$). NADH is the principal electron acceptor in glycolysis, which results in two NADH molecules per glucose molecule. The Krebs cycle also reduces ${\mathrm{NAD}}^{+}$ to NADH in three of its reactions. During oxidative phosphorylation, NADH is oxidized to ${\mathrm{NAD}}^{+}$ by donating electrons to the electron transport chain, and these electrons are ultimately accepted by oxygen.^8^^,^^9^ In the case of anaerobic glycolysis, ${\mathrm{NAD}}^{+}$ is converted to NADH and oxidative phosphorylation is diminished, which creates an overall increase in NADH abundance. Thus, the reduction–oxidation pair $\mathrm{NADH}/{\mathrm{NAD}}^{+}$ serves as an indicator of balance between oxidative phosphorylation and glycolysis. Flavins such as FAD are also involved in cellular oxidation–reduction reactions. The reduced form (${\mathrm{FADH}}_{2}$) is oxidized to FAD in complex II of the electron transport chain, while FAD is reduced to ${\mathrm{FADH}}_{2}$ in pyruvate decarboxylation and the Krebs cycle.

NADH and FAD are fluorescent while ${\mathrm{NAD}}^{+}$ and ${\mathrm{FADH}}_{2}$ are not. The fluorescence of NADH and NADPH are difficult to distinguish, and their combined fluorescence is referred to as NAD(P)H. Due to the pivotal role of NADH, NADPH, and FAD in cell biology and metabolism, these endogenous fluorophores have been used to monitor cellular redox reactions, energy metabolism, and mitochondrial anomalies under different pathophysiological conditions. Chance and others in the 1980s established NAD(P)H and FAD fluorescence for metabolic imaging.^44^^–^^48^ The use of endogenous fluorescence enables noninvasive metabolic imaging of cells and tissue in their native physiological environment without perturbations associated with contrast agents. After the development of FLIM instrumentation, biophysicists began to relate the fluorescence lifetimes of NAD(P)H and FAD to cellular metabolism.^24^^,^^25^^,^^29^ The fluorescence lifetime of NAD(P)H is significantly shorter in the free state (∼400  ps) compared with the protein-bound state (∼1 to 5 ns) of the molecule.^24^^,^^25^^,^^27^ This is due to quenching in the free state as the NAD(P)H molecule folds and diminished quenching in the protein-bound state as the NAD(P)H molecule extends. Conversely, FAD has a longer lifetime in its free state (2.3 to 2.9 ns) compared with its protein-bound state (<0.1  ns).^29^^,^^30^^,^^49^^,^^50^ Bird et al. used FLIM to demonstrate a correlation between the redox ratio ($\mathrm{NADH}/{\mathrm{NAD}}^{+}$) and the relative amounts of free to protein-bound NAD(P)H.^51^

### 1.3 FLIM-FRET Microscopy

**Fig. 2:**
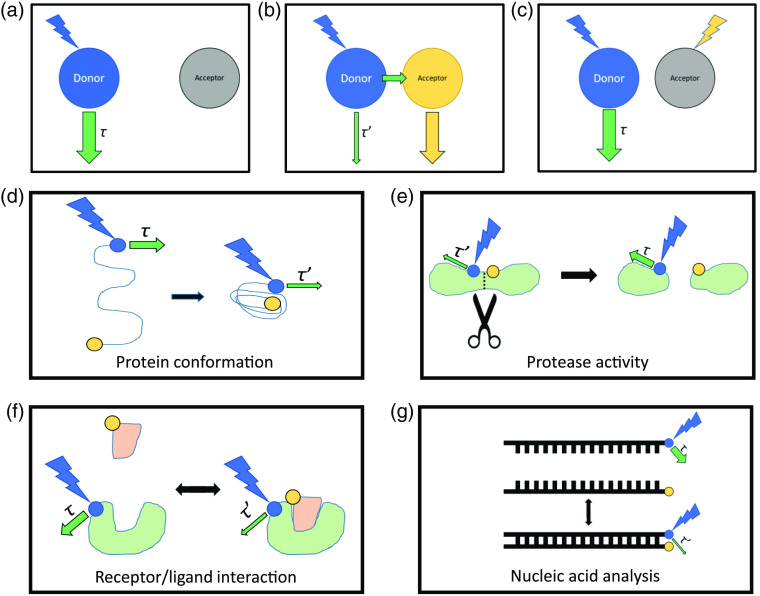
FLIM-FRET concept and applications. (a) At the Förster distance (R_0_, defined by the specific donor–acceptor pair), the efficiency of energy transfer between donor and acceptor is 50%, such that large distances exhibit low efficiencies of energy transfer. At these large distances, the fluorescence lifetime of the donor (τ) is not affected by FRET. (b) As the distance between donor and acceptor decreases, FRET can occur, quenching the emission of the donor and shortening the donor lifetime decay ($\tau^{\prime }$). (c) Photobleaching of the acceptor can confirm that the change in donor emission lifetime was due to a FRET interaction. (d), (e) Donor and acceptor pairs within the same molecule can be used to detect changes in (d) protein confirmation or (e) cleavage of proteins by proteases. (f), (g) Donor and acceptor pairs in separate molecules can be used to detect (f) receptor/ligand binding and (g) hybridization or splitting of nucleic acid strands.

The fluorescence lifetime of a donor fluorophore changes when it undergoes FRET with an acceptor molecule. As a result, FLIM can visualize changes in the proximity of FRET pairs.^17^^,^^18^^,^^52^ Specifically, the quenching of the donor emission by FRET leads to a decrease in its lifetime. FRET has been used to detect conformational changes within proteins, receptor/ligand interactions between proteins, hybridization or splitting of nucleic acid strands, membrane lipid interactions and distributions,^16^ the activity of proteases, chromatin architecture,^53^ and many other phenomena. Genetically engineered FRET pairs can be strategically expressed in biological systems for any application in which distances between proteins or protein subdomains are of interest (Fig. 2). A detailed review of FRET can be found elsewhere.^54^

FLIM-FRET has a number of advantages over intensity-based FRET. In addition to the advantages of FLIM over intensity imaging that will be discussed in Sec. 1.4, there are also benefits specific to FRET interactions. Most importantly, FLIM-FRET only requires the measurement of the donor lifetime, so direct excitation of the acceptor is not needed and acceptors with poor quantum efficiencies can be used. In addition, less excitation intensity is required for FLIM-FRET because wider emission filters can be used, allowing for FLIM-FRET pairs that are less photostable. ^55^ A portion of the donor fluorophores can fail to excite in some FRET experiments, which introduces additional errors in intensity-based FRET that are avoided in FLIM-FRET. Finally, FLIM-FRET and multiexponential fitting can be used to quantify the proportion of quenched and unquenched donor molecules.^18^ FRET events can be confirmed by photobleaching the acceptor, which should result in a donor lifetime at pre-FRET levels. The drawbacks of FLIM-FRET versus intensity-based FRET mirror those of using FLIM in general with the additional stipulation that a carefully measured reference lifetime value for the donor alone (without acceptor present) is needed for accurate calibration.

A number of considerations need to be made to select FRET pairs (FPs) that are specifically suited for use with FLIM-FRET (Table 2). Some pairs that are not useful for intensity FRET are quite useful for FLIM-FRET. This is because intensity FRET requires spectral overlap between donor and acceptor, while FLIM-FRET is optimized using pairs with well-separated emission spectra.^55^^,^^69^ A donor fluorophore will ideally have a long, monoexponential decay. With simple decay kinetics, it is easier to determine the distinct lifetime of the quenched donor using multiexponential fitting of the decay data.^55^ The unquenched single lifetime of the donor should also be as long as possible to optimize the dynamic range of the FP. Donors should also have high photostability and should not photoconvert, which could lead to an overestimation of quenching. Acceptors should have a high absorbance coefficient but an extremely low quantum yield to avoid acceptor emission in the donor channel. By choosing an optimal acceptor, donor emission can be collected using a wider spectral window to increase signal while reducing excitation power. In addition, another probe could be added to the acceptor spectral window to correlate FRET interactions with the behavior of another labeled protein.^55^

**Table 2:** Examples of FRET pairs for FLIM-FRET imaging.

Donor	Acceptor	Reference
mCerulean3	YFP	56
mTurquoise	YFP	56, 57
NowGFP	tdTomato	58
NowGFP	mRuby2	58
Clover	mRuby2	59
TagRFP	mPlum	60
mEGFP	ShadowG	61
mEGFP	mCherry	62, 63
mEGFP	mRFP1	64
mTFP1	EYFP	65
mEGFP	REACh	66
mEGFP	sREACh	67
mEGFP	ShadowY	68

New approaches to improve the FLIM-FRET continue to emerge. One goal of recent efforts is fast FLIM-FRET to capture rapid cellular events, increase imaging throughput, and quickly acquire volumes of three-dimensional (3-D) biological interactions. For example, Poland et al.^70^ developed a multifocal multiphoton system that simultaneously acquires multiple planes of FLIM-FRET using an array of beamlets produced by a spatial light modulator. Other techniques employ many parallel detectors to rapidly image protein–protein interactions in live cells.^71^ Recent developments in FLIM-FRET analysis techniques provide highly localized information on molecular interactions. Phasor analysis (see Sec. 3.1.2) of FLIM-FRET data has quantified chromatin organization at the nucleosome level, which is below the diffraction limit of most imaging modalities.^72^ Here, increased FRET between fluorescent histones signals an increase in nucleosome proximity.

### 1.4 Advantages of FLIM Over Intensity Imaging

**Fig. 3:**
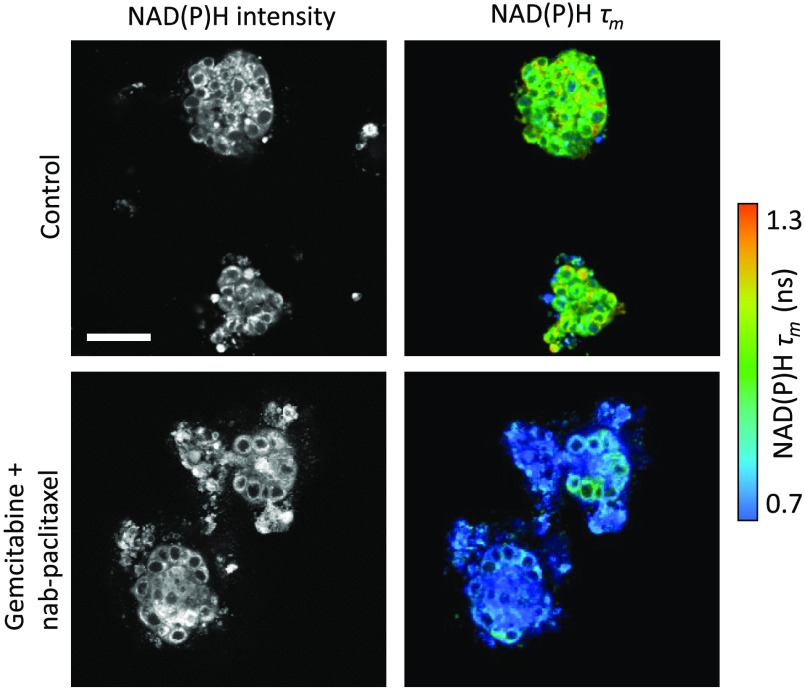
FLIM provides metabolic contrast in 3-D tumor organoids treated with chemotherapy. The $\tau_{m}$ of endogenous NAD(P)H is sensitive to the metabolic response to chemotherapy in patient-derived pancreatic cancer organoids. NAD(P)H intensity measurements alone did not distinguish treatment. Here, $\tau_{m}$ is calculated from a two-exponential decay of the free and protein-bound lifetimes of NAD(P)H. Scale bar=50  μm. Adapted with permission from Ref. 73.

FLIM offers many unique advantages over intensity-based fluorescence microscopy. Fluorescence intensity imaging provides information on the spatial distribution of fluorophores and can discriminate between fluorophores with distinct spectral properties. However, intensity alone cannot distinguish fluorophores with similar spectra or distinguish unique molecular environments around the same fluorophore. FLIM can frequently discriminate spectrally overlapping fluorophores using the fluorescence lifetime. For example, NAD(P)H often appears indistinguishable in different cellular environments based on fluorescence intensity and spectral information, but it can be distinguished easily using FLIM (Fig. 3). Overall, FLIM is advantageous in its ability to detect changes in the molecular environments of fluorophores to provide information about fluorophore function and behavior that could not be elucidated with intensity measurements alone.^74^

Unlike intensity-based measurements, FLIM is largely independent of fluorophore concentration. This means that FLIM can determine whether a change in fluorescence intensity is due to changes in quantum yield (e.g., fluorescence quenching), a variation in the overall concentration of the fluorophore, or both. FLIM measurements are also less vulnerable than intensity measurements to inner filter effects, which are absorption and scattering events that modulate the detected fluorescence intensity. Therefore, FLIM is well-suited for accurate measurements of quenching dynamics.^75^^,^^76^ Multiple configurations or states of a fluorophore can be detected with FLIM at a single location or pixel. For example, both bound and unbound fluorophores, as well as proteins with distinct folding states, will have different molecular environments that coexist within the same pixel.

FLIM is a self-referenced measurement (i.e., independent of absolute detected intensity), so FLIM experiments do not require the throughput calibration steps that are needed for intensity-based experiments. Lifetime is an absolute measurement that can be repeated across numerous device configurations (e.g., excitation intensity, detector sensitivity, and path length) after accounting for the instrument response function (IRF) of that device. Thus, artifacts caused by nonuniform illumination, which would greatly affect intensity measurements, are mitigated by measuring the lifetime. This internal calibration has the added advantage of making FLIM experiments more reproducible and comparable between different instrumentation configurations. In addition, lifetime measurements are independent of excitation and emission light scattering in cells and tissues, provided that any time delay is smaller than the resolution of the timing electronics. In addition, confounding scattering profiles can be modeled in a fitting routine by assuming a Gaussian spread of the IRF function. This is conventionally used in commercial time correlated single photon counting (TCSPC) packages such as SPCImage^77^ (Becker & Hickl), allowing FLIM to be performed accurately at deeper penetration depths.

The key downsides to time-resolved measurements include a long acquisition time that may prevent visualizing fast events, requirements for time-resolved electronics and accurate IRF measurements, and sensitivity to changes in temperature, pH, and viscosity that complicate data interpretation. Promising new techniques have increased the speed of FLIM, which should enable visualization of fast dynamics in the future.^78^^–^^80^

## 2 Instrumentation

FLIM measures the fluorescence decay rate of a fluorophore on the timescale of subnanoseconds to hundreds of nanoseconds. For reference, light travels at a speed of $3\times 10^{8}\;m/s$ or ∼1 foot in 1 ns. Fast electronics coupled with efficient photon detectors have been integral tools for FLIM and other fast temporal measurements.

**Fig. 4:**
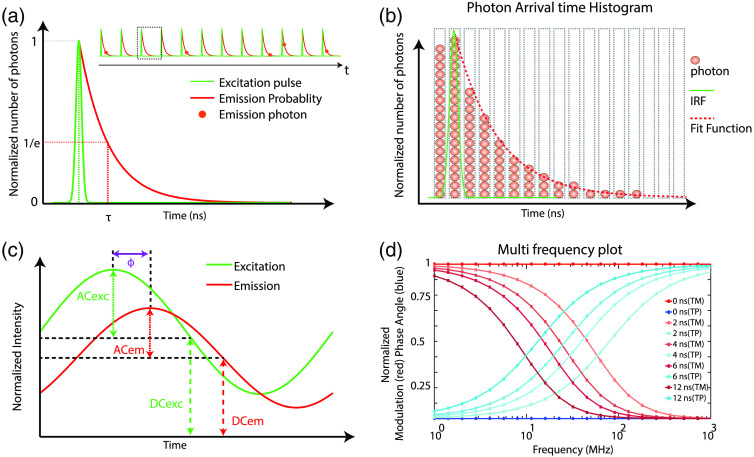
Schematic of time-domain (TCSPC) and frequency-domain FLIM. (a) TCSPC FLIM acquisition includes the short excitation pulse, single exponential fluorescence decay curve, and lifetime (τ) defined at the 1/e value. Inset shows detected single fluorescence photons (red circles) at different time periods within multiple excitation pulses. (b) Photon time of arrival histogram built from the detection time of multiple fluorescent photons (red circles); green line represents the IRF, and dotted red line represents the fit function. (c) Schematic diagram of frequency-domain measurement with sinusoidally modulated excitation (exc) and the resulting phase shifted emission (em) signal. The AC and DC components of each signal are also indicated. (d) Modulation and phase versus frequencies for different lifetimes. TM, modulation lifetime; TP, phase lifetime.

Time-domain and frequency-domain FLIM measurements are overviewed in Fig. 4, with detailed descriptions below. Briefly, time-domain fluorescence lifetime measurements use a short pulse of light for excitation (short relative to the lifetime of the sample) and then record the exponential decay of fluorescent molecules either directly (i.e., by gated detection or pulse sampling) or using time-resolved electronics that bin photons by their arrival times [Figs. 4(a) and 4(b)].^74^^,^^81^^–^^84^ Alternatively, frequency-domain techniques can also measure fluorescence lifetimes [Figs. 4(c) and 4(d)].^85^^,^^86^ Here, the excitation is continuous with amplitude modulation over time as a sine wave. The fluorescence signal shifts in phase and amplitude with respect to the excitation wave. The phase delay and amplitude modulation for a fluorophore are visualized by plotting the phase changes over a range of modulation frequencies [Fig. 4(d)]. This resulting fluorescence sine signal can be demodulated in the frequency-domain to quantify the delay induced by the exponential decay of the fluorescence intensity.

The most common implementation of FLIM is with a fast electronic method called TCSPC [Fig. 4(a)]. In TCSPC, a fast stop-watch measures the time between an excitation photon and emission photon. This time defines each emission photon’s time-of-arrival. The fast clock time is experimentally measured with a time–amplitude converter circuit (TAC), which converts the photon time-of-arrival to an analog voltage that can be recorded. In conventional TCSPC, at high photon count rates, most of the incoming photons will not be measured due to the instrument dead time. This will lead to the pile-up effect where only the photons with shorter arrival times will be recorded per excitation pulse. This loss of photons with longer arrival times will create an incorrect photon histogram, leading to overall shortening of the measured fluorescence lifetime. To avoid these effects, a low photon count at the detector is desirable, ideally <10% of the excitation repetition rate. Thus, in general, time-domain methods detect one fluorescence photon across several excitation pulses, so many excitation pulses are required to build a histogram [Figs. 4(a) and 4(b)]. The signal-to-noise ratio (SNR) of FLIM measured by photon counting, (${\mathrm{SNR}}_{\mathrm{FLIM}}$), depends on the number of photons detected per pixel (N), such that it changes with the square root of N:^87^

$${\mathrm{SNR}}_{\mathrm{FLIM}}\propto \sqrt{N}.$$ (4)

Therefore, to improve the ${\mathrm{SNR}}_{\mathrm{FLIM}}$, the photon detection process is repeated thousands of times to generate a distribution of time-of-arrivals of fluorescence photons, which is the measured exponential fluorescence decay. This improves accuracy of FLIM data analysis by curve fitting, which will be discussed in Sec. 3.

Practically, TCSPC employs an efficient method known as reverse-TAC to measure the time between the emission photon and the next excitation photon. After the histogram is made with reverse-TAC, the time axis is inverted. Reverse-TAC mode is advantageous over the forward-TAC mode in systems with high repetition rates, such as laser sources in the range of 50 to 100 MHz, but with low photon count rates. In reverse-TAC mode, TAC is reset only when a photon is detected, using the reset signal from the consequent laser pulse, thus avoiding the requirement of additional TAC reset circuits. The measured decay is a convolution of the excitation pulse and fluorescence decay [Figs. 4(a) and 4(b)]. For an ideal delta excitation pulse, the measured fluorescence decay would equal the actual fluorescence decay. The alternative to TAC is a time–digital converter (TDC), which converts the time to a digital value of delay. Experimentally, both TAC and TDC are realized using a programmable logic gate array called a field programmable gate array (FPGA), but TAC and TDC use different electronic means to calculate the time delay between the excitation photon and detected photon. TAC systems are often credited with higher quality timing due to reduced timing jitters (error in timing estimation). Photon detection is usually achieved with a constant fraction discriminator (CFD) circuit to read the analog voltage output from the detector. This discriminator circuit determines the photon counts and triggers the stop clock for TCSPC. These methods are discussed in detail in previous publications.^74^^,^^82^

One of the main limitations of TCSPC is slow acquisition speed, which motivates new techniques to expedite FLIM imaging. TCSPC and other FPGA-based architectures have long dead times (tens of nanoseconds) between photon detection events.^74^^,^^88^ Therefore, recent adoption of high-speed digitizers (subnanosecond sampling) aim to decrease dead times to two or less nanoseconds between photon detection events, so fluorescent decays can be generated more rapidly. These digitizers have been used for FLIM of endogenous and exogenous fluorophores in cells and tissues^78^^–^^80^ and offer great promise for shorter FLIM acquisition times. Other strategies to increase speed focus on minimizing dead times, parallelizing TCSPC, and implementing TCSPC with multifocal excitation.^88^^–^^94^ A detailed discussion on challenges and current approaches to improve FLIM can be found in a previous review.^95^

Other time-domain methods include time-gating (TG) and pulse sampling. In TG FLIM, following a short excitation pulse, the fluorescence decay is directly sampled at two or more time gates that are sequentially delayed from the excitation pulse.^96^^–^^99^ For a single exponential decay, the lifetime (τ) can be calculated using two equal time gates at Δt time separation^83^^,^^100^

$$\tau =\Delta t/\ln(I_{1}/I_{2}),$$ (5)

where $I_{1}$ and $I_{2}$ are the intensities measured at the two gates, respectively. For multiple fluorophores, however, two time gates would yield only a mean lifetime. Thus, multiple precisely synchronized gates, in combination with decay analysis techniques such as exponential fitting and phasor approach, are employed for multiexponential lifetime calculation.^83^^,^^98^^,^^101^^,^^102^ Following the first demonstration of multiphoton laser scanning TG FLIM by Sytsma et al., the technique has been employed in multiple studies.^103^^–^^105^ The TG approach has been more widely adopted for time-domain wide-field FLIM and will be discussed in Sec. 2.1.1. TG is advantageous over TCSPC because the electronics dead time is significantly lower (about 1 ns) and the high count rates result in faster acquisition times.^98^ However, TG lacks the sensitivity and time resolution of the TCSPC approach.^106^^,^^107^ Moreover, limitations in the number of gates and counters might result in undersampling of the decay.^87^ Another consideration is the convolution of the IRF with the intensity measured at the first time gate. This can be solved by either correcting for the IRF or delaying the first time gate after the IRF. However, this delay causes additional loss in photon efficiency.^87^ For a detailed discussion on TG FLIM and comparison with TCSPC, please refer to prior publications.^98^

Similar to TG, the time-domain pulse sampling approach involves direct measurement of the decay signal after a short excitation pulse; it was first demonstrated by Steingraber and Berlman in 1963.^108^ The fluorescence signal is continuously measured by a fast response detector and sampled by fast digitizers.^84^^,^^106^ The decay is reconstructed from all detected photons for a single excitation. The time-domain approach was traditionally employed for nonimaging point measurements of time-resolved fluorescence spectroscopy.^109^^–^^112^ Recent developments include pulse sampling coupled with optical fiber-based multispectral fluorescence lifetime imaging (FLIm).^113^^–^^117^ The fast acquisition speeds make this approach attractive for spectroscopy in clinical applications. Furthermore, the measured fluorescence signal is not affected by background light, allowing data acquisition in a clinical setting including operating rooms without the need to dim or turn off the room light.^11^^,^^116^ It has also been implemented with other imaging techniques such as optical coherence tomography^118^ or ultrasound^119^ for bimodal imaging. Current implementation of FLIm includes microchannel plate (MCP) detectors and a high-speed (12.5  GS/s) digitizer.^116^ In a recent development, a second pulsed laser was multiplexed in time with the excitation laser traditionally used for endogenous fluorophores. This allowed additional exogenous fluorophores to be imaged.^117^ MCPs have a high response time but low gain; thus they perform better with samples with high quantum efficiencies.^74^^,^^106^ The pulse sampling technique also suffers from uncertain accuracies in the lifetime decay curve reconstruction since the instrument noise characteristics are unknown.^106^ Pulse sampling techniques are discussed in detail in prior publications.^84^

Frequency-domain lifetime measurements date back to 1927 when, for the first time, lifetime was measured by Gaviola.^120^ Venetta in 1959 demonstrated lifetime measurements by coupling a phase fluorometer to a microscope, one of the seminal works leading to present day FLIM.^121^ In frequency-domain FLIM, the sample is excited by an amplitude modulated light source at high frequencies (MHz), and the harmonic response of the system is measured [Fig. 4(c)].^122^ The equation of this modulated excitation signal for a given frequency of modulation can be written as^123^

$$E(t)=E(0)[1+M_{E}\;\sin(\omega t)],$$ (6)

where E(t) is intensity at time t and E(0) is intensity at time=0. $M_{E}$ is the excitation modulation factor, and ω is the angular frequency and is given by ω=2πf, where f is the linear modulation frequency. With a sinusoidal excitation, the emission signal will also be modulated sinusoidally.^122^^,^^124^ However, the emission signal will be phase shifted with respect to the excitation due to delay between the absorption and emission. This can be written as

$$\;F(t)=F(0)[1+M_{F}\;\sin(\omega t+\phi )],$$ (7)

where F(t) is the fluorescence intensity at time t and F(0) is that at time t=0.^124^ $M_{F}$ is the emission modulation factor, and ϕ is the phase delay between excitation and emission. The modulation and phase shift of the emission is dependent on the relative values of the frequency of modulation, f, and lifetime τ [Fig. 4(d)]. In the case of single exponential decays, the phase lifetime ($\tau_{P}$) is

$$\tan\;\phi =\omega \tau_{P}.$$ (8)

The modulation factors can be expressed as the ratio of AC to DC components of the respective excitation (EX) and emission (EM) signals

$$M_{E}=\frac{{\mathrm{AC}}_{\mathrm{EX}}}{{\mathrm{DC}}_{\mathrm{EX}}}\;M_{F}=\frac{{\mathrm{AC}}_{\mathrm{EM}}}{{\mathrm{DC}}_{\mathrm{EM}}}.$$ (9)

From this, we can estimate the relative modulation M as

$$M=\frac{{\mathrm{AC}}_{\mathrm{EM}}}{{\mathrm{DC}}_{\mathrm{EM}}}/\frac{{\mathrm{AC}}_{\mathrm{EX}}}{{\mathrm{DC}}_{\mathrm{EX}}}.$$ (10)

The relative modulation (M) and modulation lifetime ($\tau_{M}$) are related by

$$M=\frac{1}{\sqrt{1+{\left(\omega \tau_{M}\right)}^{2}}}.$$ (11)

In the case of a single exponential decay, lifetimes from phase and relative modulation are equal, (i.e., $\tau_{P}=\tau_{M}$ for all ω). However, for multiexponential decays, $\tau_{P}<\tau_{M}$ and absolute values will depend on the modulation frequency.^123^ The angular modulation frequency should be set to roughly the inverse of the lifetime (i.e., ωτ=1) to give maximum sensitivity. Linear modulation frequencies of ∼100  MHz to 1 GHz give picosecond temporal resolution, which is appropriate for fluorescence lifetime measurements. Given these constraints on modulation lifetimes in frequency-domain measurements, phase lifetimes are preferred to modulation lifetimes.

One major advantage of frequency-domain FLIM over time-domain FLIM techniques, such as TCSPC, is acquisition speed, making frequency-domain an ideal technique for measuring rapid cellular events. The slower processing electronics used in TCSPC can also limit the ability to accurately measure lifetime in very bright samples with high photon count rates. Previously described TG FLIM, pulse sampling techniques, and new faster TAC/TDC electronics (2 to 100 ns dead time) have improved current time-domain FLIM acquisition times, bringing them closer to frequency-domain (0 ns electronics dead time). One of the latest advancements includes implementation of frequency-domain FLIM in a multiphoton microscope capable of imaging deeper than conventional systems.^125^ Finally, frequency-domain FLIM can be implemented without the use of costly pulsed lasers.

On the other hand, TCSPC can provide better timing resolution and higher SNR for weakly fluorescent samples due to its ability to time individual photons. Thus, frequency-domain may be more advantageous for brighter, more dynamic samples, while TCSPC may be beneficial for weakly fluorescent, static samples. These dynamic range limitation errors are pronounced for fitting routines that use spatial binning for increasing accuracy, when the fitting would automatically be biased by the larger number of photons from neighboring pixels. In addition, since individual photons are timed, TCSPC can distinguish between individual components of a multiexponential decay with high accuracy. To resolve multiple components in the frequency-domain, the signal must either be recorded using multiple modulation frequencies^123^^,^^126^ or digital heterodyning aided with phasor analysis techniques.^127^^,^^128^ This is summarized in Table 3.

**Table 3:** Advantages and limitations of time and frequency-domain FLIM techniques.

FLIM technique	Advantages	Disadvantages	References
TCSPC	• High accuracy of lifetime estimation	• Requires costly pulsed lasers	85, 87
		• Poor performance with high photon count rates or dynamic samples	
	• Provides better SNR for weakly fluorescent samples		
	• Estimates multiple lifetime components		
Time gating	• Lower electronic dead time than TCSPC electronics	• Low sensitivity and time resolution	87, 98, 106
	• High count rates (no count rate limitation)	• Poor performance with low photon counts	
	• Fast acquisition speed	• Undersampling of decay signal due to limited gates and counters	
Pulse sampling	• Fast acquisition speed	• Requires costly electronics and pulsed laser	11, 74, 84, 106, 116
	• Relatively unaffected by background light		
		• Low gain of detector (MCP)	
		• Instrumentation noise characteristics unknown	
Frequency domain	• Fast acquisition speed	• Poor performance with low photon counts	85, 87
	• Performs well with bright samples		
	• Can be implemented without pulsed lasers		

### 2.1 Microscopy

**Fig. 5:**
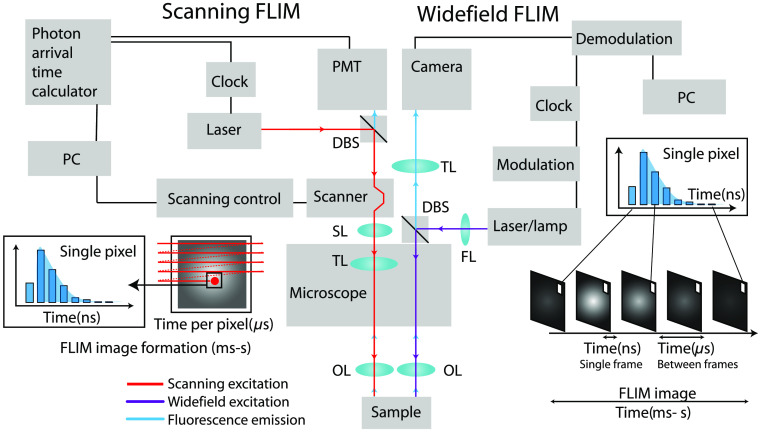
Schematic showing FLIM implementation in scanning and wide-field configurations. Two imaging modalities are compared side by side: scanning TCSPC FLIM and wide-field TG FLIM. The scanning beam of laser light from a galvanometric mirror is projected onto the back focal plane of the objective lens (OL) using the scan lens (SL) and tube lens (TL). The size of the beam and scan angle is often adjusted by varying the SL-TL pair. The light from the back focal plane is then focused by the objective lens into an excitation light cone. The emission from the same light cone is retraced back through a dichroic beamsplitter into a photon detector unit such as a photomultiplier tube (PMT). The electrical current from the PMT is amplified and read by an electronic board to calculate photon arrival times. These photon times are linked to the pixel of illumination by the computer (PC) that controls the scanner position in the image and thereby produces a histogram of photon arrival times for each pixel as shown in the inset of the left. LSM FLIM typically achieves 4 to 10 frames per second (fps) acquisition speed, which is usually limited by the scanner speed. (Right) Wide-field FLIM requires a focusing lens (FL) to achieve a field of illumination. The fluorescence from the focus of the objective lens is magnified by a tube lens and then imaged onto a camera sensor. FLIM in wide-field systems is achieved using a short frame exposure time (ns per frame). However, wide-field FLIM requires repeated frame acquisitions over a total time of milliseconds to seconds to collect sufficient photons for a complete histogram of fluorescence decay, as shown in the inset on the right side. Recent FLIM cameras intelligently select modulation-demodulation waveforms to achieve faster FLIM frame rates of ∼15  fps.

Two microscopy imaging schemes are used for FLIM: laser scanning microscopy (LSM) and wide-field illumination (WFI) microscopy. LSM and WFI are compared in the FLIM imaging schematic shown in Fig. 5. LSM systems are subdivided based on their excitation-detection method as either confocal (CLSM) or multiphoton (MP-LSM). All of these microscopy techniques offer optical sectioning, which allows 3-D FLIM imaging. However, it is important to note that certain clinical applications such as surgical guidance, endoscopy, ophthalmoscopy, and others do not always require sectioning and can work in a topological imaging modality or a lensless imaging scheme.^116^^,^^129^ FLIM imaging optics require a light source for illumination and sensitive detection to distinguish the photons of interest from background photons. However, the key component of FLIM is the electronics used to estimate the timing of detected photons within each pixel. Numerous strategies are in development for more sensitive detection methods.^130^^–^^134^

#### 2.1.1 Wide-field FLIM

WFI uses a parallel illumination field at the focus of the objective lens and collects fluorescence from the focal plane onto a camera (Fig. 5). Wide-field FLIM is often used for rapidly imaging large sample areas since light from the entire field of view is collected using a camera-based detection. Wide-field FLIM uses either time-domain techniques, such as TCSPC^135^ or TG, in which a series of fluorescence images are collected by shifting a timing window (order of nanoseconds) through the emission decay,^136^^–^^138^ or frequency-domain methods of demodulating the fluorescence signal from the excitation frequency.^139^

Wide-field FLIM has the advantages of higher frame rates and less photodamage when compared with LSM. However, camera sensitivity and SNR are not as high as that of LSM detectors, which results in poorer axial resolution. In wide-field collection, every camera pixel simultaneously detects scattered light from all other pixels of the illuminated area, which intermixes the timing-spatial coordinates.^133^ Assuming a fixed photon emission rate from the sample, image optimization is a trade-off between either spatial or temporal resolution.^133^ Wide-field techniques such as structured illumination and spinning-disk confocal can achieve higher spatial resolution without compromising imaging speed.^136^^,^^140^^–^^143^ Other advantages of wide-field systems are their simpler implementation and the low computational cost to assign photon detection times in each pixel. Some of the advancements in wide-field FLIM include its implementation with Nipkow disc microscopy for fast 3-D FLIM imaging,^142^ wide-field coupled with single plane illumination microscopy for high-resolution 3-D FLIM,^144^ TG single photon avalanche diode (SPAD) cameras for phasor-based high speed wide-field FLIM,^145^ multifrequency widefield,^146^^,^^147^ and image gating by pockel cells.^148^ Current wide-field FLIM systems are discussed in detail by Suhling and Hirvonen et al.^149^

#### 2.1.2 Laser Scanning Microscope FLIM

Laser scanning microscopes have out-of-focus rejection methods that enable higher contrast and spatial resolution than wide-field systems. In comparison with wide-field FLIM, LSM-FLIM modalities are often coupled to faster electronics to generate precise photon detection times per pixel. As discussed previously, these methods fall under two modalities of timing calculation: TDC-based or TAC-based.

#### 2.1.3 Laser sources

FLIM uses modulated laser sources for illumination. This can be achieved by many modern pulsed laser diodes that are modulated with an internal trigger or the high power density, ultrafast laser sources developed in the 1990s. These lasers have a remarkably short pulse duration (hundreds of femtoseconds), durable repetition frequency (in the order of 0.1 GHz), and tunable wavelengths in the near-infrared region. These lasers are currently used extensively for *in vivo* and small animal imaging due to their use as a multiphoton excitation (MPE) source (explained in the section below). Pulsed light sources are popular because of numerous applications in digital communications and remote sensing. Nonlinear light sources, such as supercontinuum sources, are also popular because they achieve near continuum tunability over a large wavelength range.

#### 2.1.4 Confocal and multiphoton microscopes

Both CLSM-FLIM and MP-LSM FLIM are broadly used in applied sciences to study biology and materials. Confocal imaging methods use a pinhole (small aperture) to reject out-of-focus light. Most biomedical confocal systems use a low power laser for excitation and focus the light at one point in space using a pair of galvanometric scanners (XY scanner). Precise movement of the objective controls for the Z position. The fluorescence emission from the 3-D focal volume retraces back through the XY scanner (thus descanned) and reaches the detector. The focused spot is scanned across the sample to detect photon density pixel-by-pixel. A computer records the photon density (i.e., fluorescence intensity) along with the location of the XY scanner and Z position to generate a CLSM image. The difference between LSM and CLSM is the use of the pinhole in CLSM that enables axial (Z-plane) selection. Comprehensive reviews of CLSM are available elsewhere.^150^^–^^152^

Multiphoton FLIM uses MPE, generally two-photon (2P) or three-photon (3P) excitation, which relies on high photon density to achieve nonlinear excitation of fluorescence. This high density is achieved by lower energy, higher wavelength photons in the near-infrared region. In 2P excitation, two photons of half the energy spontaneously come together to excite the molecule to a higher electronic energy level, which then follows its regular radiative decay (fluorescence) route to relax back to the ground state. Multiphoton FLIM is widely used for tissue imaging because near-infrared wavelengths achieve deeper penetration depths in tissues compared to the visible wavelengths that are commonly used in single photon excitation. This is due to reduced scattering and absorption in tissues within the near-infrared wavelength window. The nonlinear excitation scheme of MPE limits the fluorescence excitation to a small focal volume comparable to the confocal detection volume, but without a pinhole. This allows MP-LSM detectors to be placed in the transmission mode (or nondescanned mode), instead of descanning through the scanning optics. This nondescanned geometry enables higher detection efficiencies. Multiphoton systems use tunable, mode-locked lasers that provide ultrashort, high intensity pulses. A popular source is titanium–sapphire crystal lasers with tunability between 680 and 1100 nm. Most MP-LSM systems include pulsed sources to achieve high photon density, so additional FLIM capabilities only require the timing electronics to estimate photon arrival times. Therefore, many MP-LSM systems likely include FLIM, unlike CLSM systems that conventionally use a continuous-wave excitation source. MP-LSM systems often collect 3-D image tomograms over deeper depths than CLSM. Reviews of MP-LSM are available from Refs. 152 to 156.

Simultaneous excitation of multiple fluorophores is advantageous over sequential imaging because it minimizes FLIM acquisition times. Simultaneous FLIM of three endogenous fluorophores in addition to second harmonic generation (SHG) signals have been achieved by multiphoton wavelength mixing.^6^ Furthermore, one wavelength has been used to excite the two intrinsic fluorophores, NAD(P)H and FAD.^157^ In addition, two single-photon wavelengths have been temporally interleaved to alternately excite NAD(P)H and FAD.^158^

#### 2.1.5 Detectors

Detectors in LSM are often characterized by their sensitivity, reproducibility, quantum efficiency, photon-counting capability, narrow temporal responses, relatively fast transit time, low dark counts, and high SNR. Most LSM detectors are photomultiplier tubes (PMTs) that can be used in a photon-counting mode, which uses discriminators as described above. MCPs, avalanche photodiode, SPAD, hybrid PMTs, and SPAD arrays are also used for FLIM detection, each with merits and challenges. For example, SPAD arrays are capable of 256×256  pixels including a TDC in each pixel, so an entire FLIM image can be acquired with <100  picosecond resolution.^159^^,^^160^ However, SPAD arrays suffer from lower quantum efficiency at 460 nm (<35%) and a low fill factor (additional microlens arrays can help to effectively guide light), which results in lower photon collection efficiency.^161^ A detailed discussion on current detectors is given by Bruschini et al.^160^

Cameras with integrated FLIM capabilities have recently gained popularity. Chen et al. demonstrated wide-field FLIM using a frequency-domain CMOS FLIM camera,^162^ while Mitchell et al. implemented a frequency-domain CMOS FLIM camera in a lightsheet microscopy system.^163^ Raspe et al. developed single-image fluorescence lifetime imaging microscopy (siFLIM) with a modulated electron-multiplied-charged couple device FLIM camera capable of simultaneously recording phase-shifted images.^164^

## 3 Analysis of FLIM Data

Quantitative analysis of FLIM images provides insights into cell function, structure–function relationships, and spatial heterogeneity that are not apparent with qualitative observations of the images. Analytical tools for fluorescence lifetime estimations, image segmentation, and heterogeneity analysis are introduced in this section. Many popular methods are highlighted and summarized in Table 4, but this is not an exhaustive list of tools. Innovations in these areas are ongoing, and new FLIM analysis tools are frequently adopted from disparate disciplines.

**Table 4:** Comparison of commonly-used fluorescence lifetime estimation methods.

Lifetime estimation methods	Advantages	Disadvantages	References
Curve fitting methods			
Least squares fitting	• High accuracy for high SNR data	• Poor accuracy for low SNR data	165, 166
	• Easy to implement	• Assumes Gaussian-distributed noise	
		• Requires pixel binning	
Maximum likelihood estimation	• High accuracy for low and high SNR data	• Assumes Poisson-distributed noise	165, 167
	• Easy to implement	• Requires pixel binning	
	• Provides flexible bin widths		
Global analysis	• Applicable to time-domain and frequency-domain data	• Long computation times	168–171
	• Incorporates spatial information to improve accuracy		
Bayesian analysis	• High accuracy for low and high SNR data	• Susceptible to error from initial assumption of decay parameters	172–174
	• Adaptable for fit-free analysis		
		• Long computation times	
	• Avoids pixel binning		
Phasor methods			
Phasor analysis	• Fit-free	• Poor accuracy for low SNR data	172, 175, 176
	• Intuitive representation of lifetime estimates		
		• Susceptible to error from instrument response	
	• Useful for large time-domain or frequency-domain datasets		
	• Visualizes lifetime heterogeneity		
Deconvolution methods			
Stretched exponential analysis	• Rapid fitting procedure	• Poor fitting for decays greater than three components	177, 178
	• No prior assumption of the decay distribution	• Requires prior knowledge of fluorescent species present	
	• Visualizes lifetime heterogeneity		
Lifetime moment analysis	• No prior assumption of IRF	• Poor accuracy for low SNR data	81, 179
		• Poor accuracy for exceptionally long lifetimes	
	• Visualizes lifetime heterogeneity		
Transformation (e.g., Fourier, Laplace)	• Rapid fitting procedure	• Poor accuracy for low SNR data	170
		• Requires increased sampling of decay curve	
Laguerre deconvolution	• Fit-free	• Poor accuracy compared with other fit-free techniques	176, 180
	• Rapid fitting procedure		
	• Precise (low variation) lifetime estimates	• Poorly suited for estimating exceptionally short or long lifetimes	

### 3.1 Fluorescence Lifetime Estimation

#### 3.1.1 Curve fitting

**Fig. 6:**
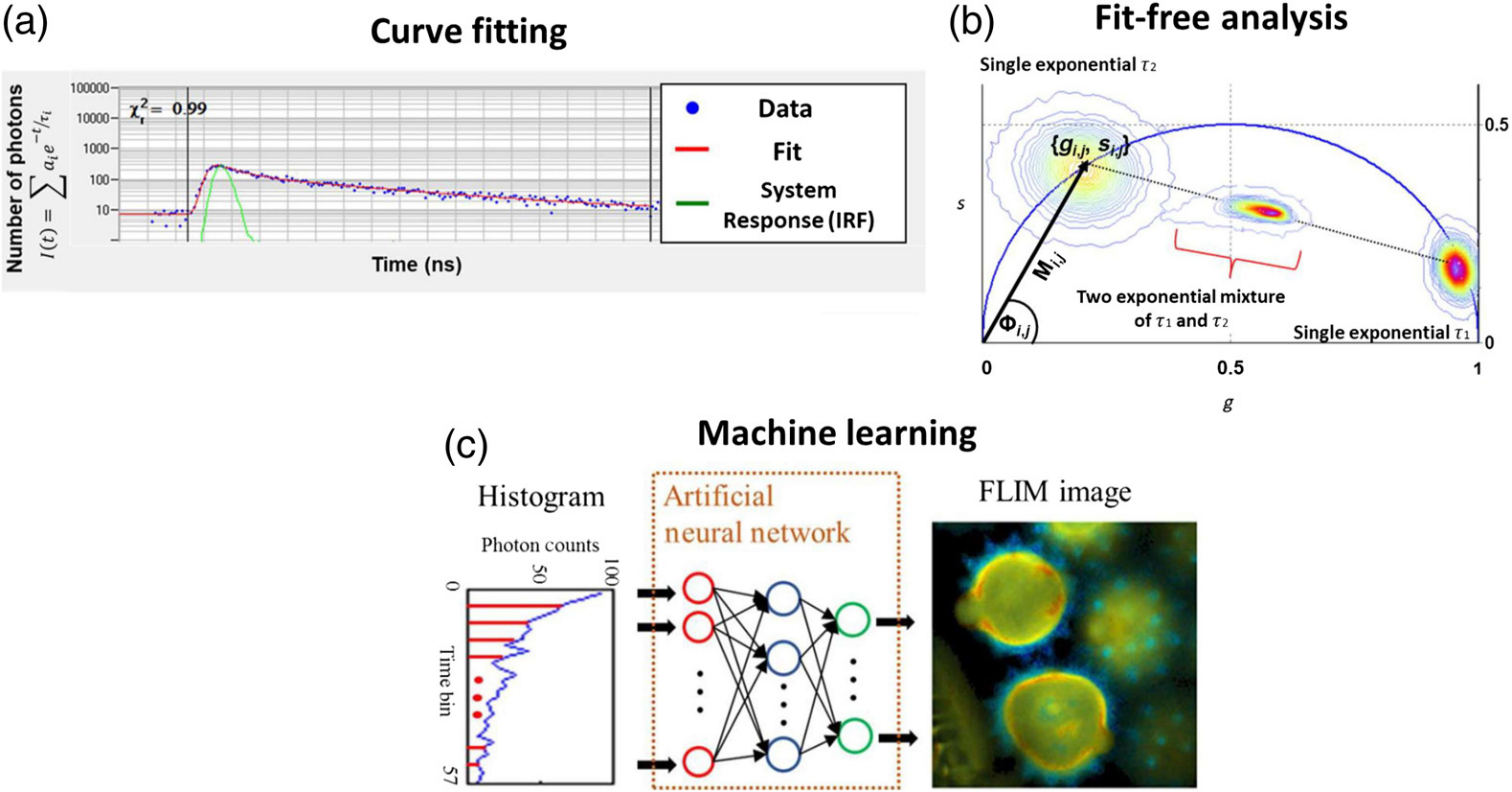
Examples of fluorescence lifetime estimation methods. (a) Curve fitting analysis determines lifetime decay variables $\left(\alpha_{i},\tau_{i}\right)$ by fitting an estimated decay function and estimated or measured IRF to experimental data. This process is iterated with the measured data to optimize goodness-of-fit parameters ($\chi^{2}$). (b) Fit-free methods for estimating lifetime parameters of time-domain or frequency-domain lifetime data also exist. The phasor approach is one such technique frequently used for intuitive representation of fit-free lifetime estimation. Here, measured lifetime data can be transformed into phasor space to visualize pixels with similar lifetime values [Eqs. (12)–(15)]. Universal circle shown as blue semicircle. Example phasors for single exponential species $\left(\tau_{1},\tau_{2}\right)$ and a two-component mixture of $\tau_{1}$ and $\tau_{2}$ illustrate the rule of linear addition. (c) Neural networks can be trained with simulated or experimental FLIM data for fast generation of fluorescence lifetime maps. Adapted under CC BY-4.0 with permission from Ref. 181.

Fluorescence lifetime measurements often capture multiple fluorophores within each pixel, resulting in multiexponential decays. In TCSPC, each photon is assigned to a time bin within a lifetime histogram.^74^ Histograms are fit to multiexponential decay functions described in Eq. (1).^76^ An IRF is measured from a sample with an instantaneous lifetime (e.g., SHG signal from a urea crystal for two-photon microscopy), which accounts for the temporal response of the optical system.^74^ Curve fitting analysis requires some prior assumptions, including the number of lifetime components, temporal offset of detected signals, and sources of background fluorescence.^74^ This method is also highly dependent on the number of photons detected per pixel as higher photon counts will improve the accuracy of the fit. The multicomponent exponential decay estimate is then convolved with the IRF and compared with the experimentally measured lifetime decay curve [Fig. 6(a)].^74^ The chi-squared ($\chi^{2}$) goodness-of-fit test is used to evaluate agreement between the fit and the measured data. Parameters of the model ($a_{i}$, $\tau_{i}$) are iterated to achieve a chi-squared value closest to 1, indicating the best model fit to the experimental data.^74^

These parameter estimates and fit quality measurements can be determined from analytical approaches, such as least squares fitting, maximum likelihood estimation, and Bayesian analysis.^165^^,^^168^^,^^172^ These methods describe the likelihood of detecting specific photon counts within each time bin from the experimental decay, based on statistical assumptions unique to each method. For example, least squares fitting minimizes the squared difference between measured fluorescence and estimated signal and assumes Gaussian noise, whereas maximal likelihood methods assume Poisson-distributed noise.^165^^–^^167^ Both approaches provide comparably accurate estimates of fluorescence decay parameters for lifetime histograms with high photon counts, though maximum likelihood analysis performs better for low photon counts.^165^ Maximum likelihood analysis also allows for varied bin widths.^172^^,^^182^

Global analysis is another approach to estimating fluorescence lifetimes from low SNR images.^168^^–^^170^ One implementation of global analysis assumes that all fluorescent species are present within each pixel.^171^ This assumption improves estimation accuracy for data with low photon counts or high background signal. Lifetime parameters of the frequency-domain or transformed time-domain data are estimated by fitting for phase shift and modulation values at multiple frequencies, as shown in Fig. 4(d).^169^^,^^171^ Alternatively, simultaneous analysis of per-pixel lifetimes assumes fixed lifetime values across all pixels and iterates lifetime parameter $\left(\tau_{i},\alpha_{i}\right)$ estimates to improve a whole-image goodness-of-fit measure.^183^^–^^185^ This approach provides better parameter estimates by conserving the spatial information typically lost from averaging photon counts across pixels.^169^ Lifetime estimates can also improve with segmentation prior to global fitting, which is important when shorter acquisition times are needed to capture fast dynamics.^170^^,^^186^

Bayesian analysis has also been used to improve lifetime estimations. This method empirically determines both the prior distribution of the fluorescence decay (not limited to Gaussian distributions) and the likelihood function^173^ to establish the posterior distribution of parameters.^172^^,^^187^ Parameter estimates are iterated to maximize the posterior distribution and provide reliable lifetime estimates.^187^ In general, this method yields optimal lifetime fits even with high noise and low total photon counts, but careful selection of the prior distribution is critical to ensuring accurate estimates.^173^^,^^187^ Recent developments in Bayesian fluorescence lifetime estimation can bypass fits to the measured data and therefore bypass assumptions of the prior distribution of lifetime parameters, which can bias the lifetime estimates.^172^^,^^174^^,^^188^

#### 3.1.2 Phasor analysis

Phasor analysis is a fit-free technique in which the fluorescence decay from each pixel is transformed into a point in two-dimensional (2-D) phasor space. Phasor representation provides a visual distribution of the molecular species in an image by clustering pixels with similar lifetimes [Fig. 6(b)]. Phasor analysis is instantaneous because it does not require an iterative fit procedure, and visualization in phasor space is especially advantageous for large FLIM datasets.^175^^,^^189^ In phasor analysis, phasor distributions corresponding to similar lifetimes (decays) can be selected to locate the corresponding pixels in the image with similar lifetimes, even if they are spatially separated.^175^

Phasor analysis can be applied to both time-domain and frequency-domain FLIM measurements. If P(i,j) represents a pixel in the FLIM image with coordinates (i,j) and $I_{i,j}(t)$ is the fluorescence intensity decay at that pixel, the corresponding coordinates in the phasor plot (g,s) for time-domain measurements are given as^190^

$$g_{i,j}(\omega )=\frac{\int_{0}^{T}I_{i,j}(t)\cos(\omega t)dt}{\int_{0}^{T}I_{i,j}(t)dt},$$ (12)

$$s_{i,j}(\omega )=\frac{\int_{0}^{T}I_{i,j}(t)\sin(\omega t)dt}{\int_{0}^{T}I_{i,j}(t)dt},$$ (13)

where ω=2πf and f=1/T is the laser repetition rate. Notably, variations in background signal or the temporal response of the optical system may introduce error into time-domain lifetime measurements transformed into phasor space, which should be considered when performing phasor transformations.^176^

In the case of frequency-domain measurements, the coordinates are given as

$$g_{i,j}(\omega )=M_{i,j}\;\cos(\phi_{i,j}),$$ (14)

$$s_{i,j}(\omega )=M_{i,j}\;\sin(\phi_{i,j}),$$ (15)

where $M_{i,j}$ is the modulation and $\phi_{i,j}$ is the phase shift of the emission signal with respect to the excitation. The phasor coordinates can also be expressed in terms of lifetime and angular laser repetition frequency (ω). In the case of a single exponential decay, the g and s coordinates are given as

$$g_{i,j}(\omega )=\;\frac{1}{1+{\left(\omega \tau \right)}^{2}},$$ (16)

$$s_{i,j}(\omega )=\frac{\omega \tau }{1+{\left(\omega \tau \right)}^{2}}.$$ (17)

From the equations of phasor coordinates, (g,s), [i.e., Eqs. (16) and (17)], the following can be derived:^123^

$$s_{i,j}^{2}+{\left(g_{i,j}-1/2\right)}^{2}=1/4.$$ (18)

From this equation, it can deduced that all single exponential lifetimes will fall on a semicircle of radius 1/2 and center (1/2,0). Since all possible single exponentials fall on this circle, it is referred to as the universal circle [Fig. 6(b)]. A short lifetime having a smaller phase will lie near the point (1,0), which corresponds to τ=0, while a long lifetime will fall near the universal circle coordinates (0,0), which corresponds to τ=∞.

Phasors follow the rule of linear addition. For example, the phasor location of a mixture of two species falls on a straight line joining the phasor location of the two individual species on the universal circle [Fig. 6(b)].^189^^,^^191^ The position on this line is determined by the relative fractional contributions of each species. Similarly, the phasor distribution of a three-exponential species will fall in the triangle formed by the three individual phasor locations and similarly for higher order exponentials.^192^^,^^193^ Hence, the phasor distribution of a heterogeneous sample will have a position inside the universal circle. This representation also provides a straightforward interpretation of the biological significance of lifetime values compared with other lifetime estimation analyses.^102^

#### 3.1.3 Deconvolution analysis

Deconvolution methods recover the lifetime decay from the measured fluorescence signal by deconvolving the estimated optical system response. Deconvolution includes variations on the least squares approach discussed above, stretched exponential/lifetime moment analysis, and methods of transformation (e.g., Fourier and Laplace transforms).^177^^,^^179^^,^^194^^–^^198^ Stretched exponential and lifetime moment methods are fundamentally similar because the lifetimes of individual species are estimated from the total measured decay distribution.^177^^,^^179^ Stretched exponential and lifetime moment methods have specific implementations for time-domain or frequency-domain data. Furthermore, Fourier and Laplace techniques transform the measured decay curve to be proportional to the product of the transformed source excitation pulse and system response.^196^^,^^197^ The lifetime and contribution of each species are then recovered from the transformed system response.^196^^,^^197^^,^^199^^,^^200^ Deconvolution methods avoid assumptions about the instrument response but still share limitations associated with standard model fitting methods.

Laguerre deconvolution is an alternative to model fitting for fluorescence lifetime estimation. Here, the measured fluorescence decay is transformed and represented in the form of Laguerre polynomials, resulting in a series expansion of the decay and convolved IRF.^176^^,^^180^^,^^195^^,^^201^ This Laguerre transformation produces linearly independent functions that enable expansion of decays with low susceptibility to noise and proportionality between fluorescence intensity and lifetime decay.^176^^,^^180^ The pixelwise linear combination of Laguerre coefficients provides the per-pixel fluorescence decay.^180^^,^^201^ The Laguerre method is less accurate but more precise in lifetime estimates compared with the similarly fit-free phasor method, especially for values at either extreme.^176^

#### 3.1.4 Machine learning analysis

Machine learning techniques are another alternative to time-intensive curve fitting procedures. A few key algorithms commonly used for this purpose are highlighted here. Simple neural networks can estimate fluorescence lifetimes directly from TCSPC data by learning weights from examples of curve fitted pixels.^181^ Variations of convolutional neural networks (CNNs) can also rapidly calculate fluorescence lifetimes. Briefly, CNNs downsample recorded images through kernel convolution and window pooling steps, resulting in a low-resolution image on which predictions of pixel class membership are made (i.e., contraction).^202^ Pixel positions from the initial pooling steps are recalled to assign class predictions to pixels in upsampled images (i.e., expansion).^202^ This computational structure has been used to analyze hyperspectral fluorescence lifetime images and to dynamically monitor fluorescence lifetimes *in vitro* and *in vivo* [Fig. 6(c)].^203^^,^^204^ Neural networks continue to improve, including simultaneous prediction of fluorescence lifetimes and object segmentation masks, which will be discussed below.

### 3.2 Fluorescence Lifetime Heterogeneity Analysis

#### 3.2.1 Pixel-level analysis

Pixel-level analysis of fluorescence lifetimes can inform on subcellular and cell-level heterogeneity within a sample. Lifetime histograms provide a useful quality check of curve fitting from TCSPC pixels, confirm the presence of distinct fluorescence lifetimes, and/or confirm expected changes in lifetime values from an experimental condition or FRET interaction.^205^ Distributions of pixels within phasor space provide complementary information on the identity of fluorophores in the sample and lifetime changes throughout an experiment.^175^^,^^205^ Pixel-level FLIM analysis has been previously used to quantify lipid membrane integrity and heterogeneity, immune cell heterogeneity, cell development, protein conformation and organization, and other phenomena.^206^^–^^211^

#### 3.2.2 Object-level analysis

Object-level analysis provides a biological context for interpreting FLIM images by averaging lifetime values across all pixels within a single object of interest (e.g., cells, organelles, and bacteria).^32^ This approach quantifies diversity across cells, organelles, and other features in a similar manner to established techniques such as flow cytometry or colony counting. Segmentation is required for object-level analysis. Automatic segmentation can be achieved with computational approaches such as multiresolution community detection and morphological filtering and thresholding.^212^^–^^214^ In addition, unsupervised clustering techniques (e.g., K-means clustering) can segment single cells and intracellular compartments for phasor-based lifetime data.^215^ Open source packages such as FLIMfit and FLIM-FRET analyzer have been developed for multiple functionalities, including automatic segmentation, lifetime decay fitting, and data visualization.^216^^,^^217^

Machine learning is also popular for image segmentation due to its high accuracy and generalization across imaging formats. Neural networks can learn features of pixels within objects to generate segmentation masks.^202^ Several architectures have been designed to improve segmentation performance, primarily for intensity-based segmentation of cellular compartments. Variations of CNNs have been developed for object segmentation. These variations include UNets, feature pyramid networks, and Mask-RCNN.^202^ UNet employs the standard CNN framework, described earlier, but maintains symmetrical contraction and expansion branches, and concatenates contracted layers with expansion layers to better preserve the initial structure of the image.^218^ Feature pyramid networks follow this scheme, but they sum convolutional layers from contraction and expansion to inform image upsampling.^219^ Similarly, mask-RCNN uses feature pyramid networks to generate feature maps for regions of interest (ROIs) before convolutional steps that classify pixels within an ROI for object masks.^220^ Modified versions of these algorithms have been used to segment numerous cell types and intracellular features across imaging platforms,^202^ and a combination of these algorithms has enabled nuclei segmentation across image types [e.g., hematoxylin and eosin (H&E) and immunofluorescence].^221^ This is not an exhaustive list of machine learning approaches for fluorescence image segmentation, but it provides an overview of some object-level segmentation and classification tools.

Segmentation of autofluorescence images is challenging due to low SNR and poor spatial specificity of the fluorescence signal. CellProfiler has been used to isolate cells, nuclei, and cytoplasms from two-photon NAD(P)H autofluorescence images.^222^ This approach identifies nuclei within a specified size range by thresholding pixels above the background fluorescence but below cytoplasmic fluorescence values. Whole cell masks are defined by propagating outward from nuclear masks. Nuclear masks are subtracted from cell masks to isolate cytoplasmic areas. Similarly, a collection of ImageJ plugins have been developed for integrated lifetime decay fitting (SLIM curve) and object segmentation (Trainable Weka Segmentation).^223^ This approach first requires input of a small subset of fluorescence images in which the user annotates the objects of interest. A number of ImageJ-based filters and transformations are applied to the images to extract features specific to annotated objects.^224^ A suite of machine learning algorithms (Weka) are then applied to annotated inputs and extracted features to classify pixels in unlabeled images.^225^ CellProfiler has developed a similar annotation-based method, Ilastik, optimized for fluorescent proteins and dyes but applicable to autofluorescence images.^226^

**Fig. 7:**
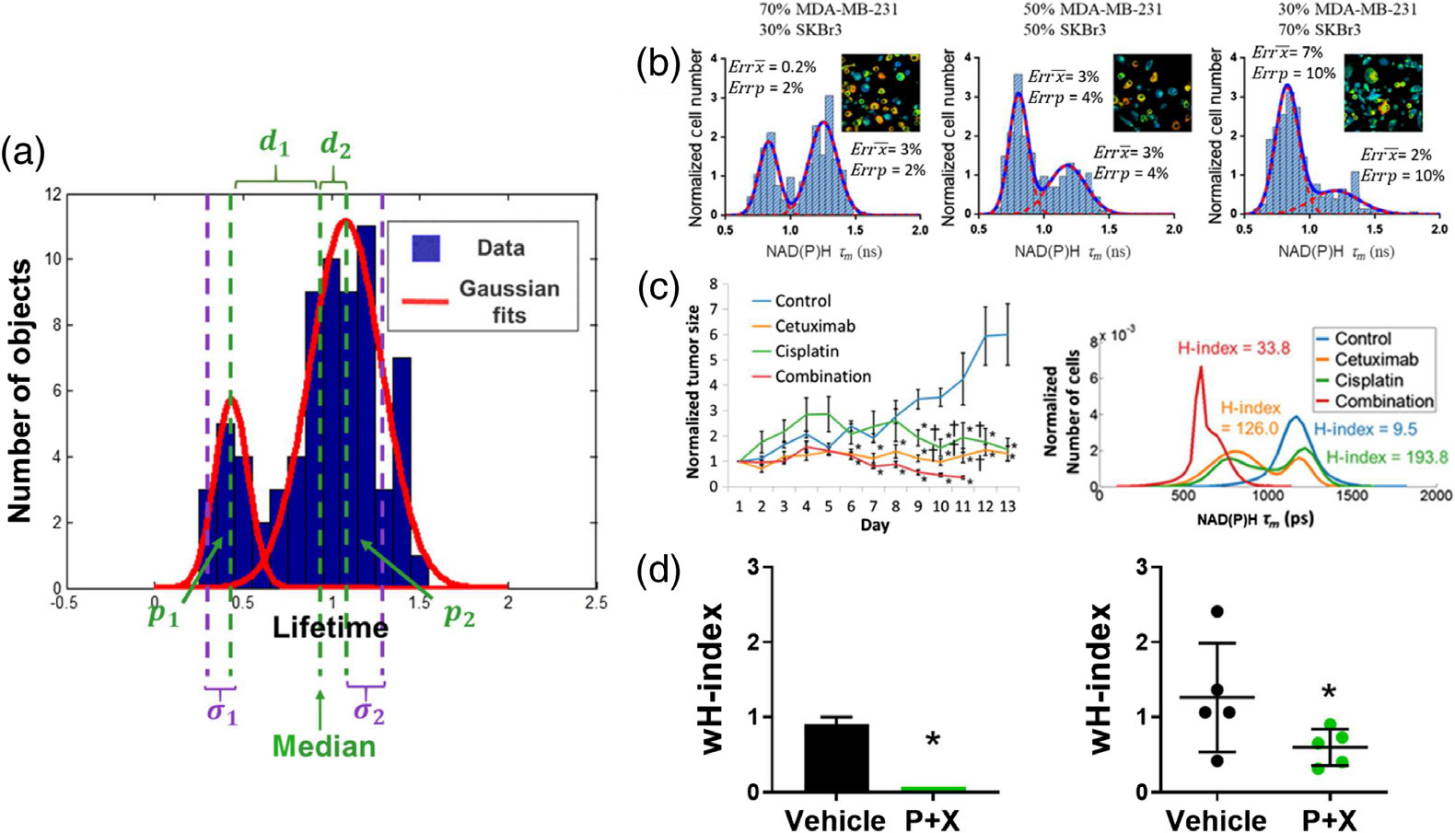
Heterogeneity analysis of fluorescence lifetime data. (a) Histograms of lifetimes per object are fit to distribution models to describe subpopulations and variability in the data. The H-index and wH-index are derived from these fits [Eqs. (19) and (20), respectively]. Here, $p_{i}$ is the proportion of each subpopulation, $d_{i}$ is the distance between subpopulation median and global median, and $\sigma_{i}$ is the subpopulation standard deviation. (b) Distribution density models fit to cell-level NAD(P)H mean lifetimes can accurately identify distinct breast cancer cell lines (MDA-MB-231 and SKBr3) in mixed cocultures (proportion of mixtures indicated above plots). Errors (Err) in the model predictions for mean ($\overset{¯}{x}$) and proportion (p) of each population are given within each plot. Adapted with permission from Ref. 227. (c) H-index of *in vivo* FaDu tumor cell NAD(P)H mean lifetime (right) correlates with *in vivo* treatment response (left). Adapted with permission from Ref. 228. (d) Cell autofluorescence wH-index is similar for (left) *in vitro* organoids derived from primary PyVmT tumors and (right) *in vivo* PyVmT tumors with vehicle and combination treatment. Adapted with permission from Ref. 229.

**Fig. 8:**
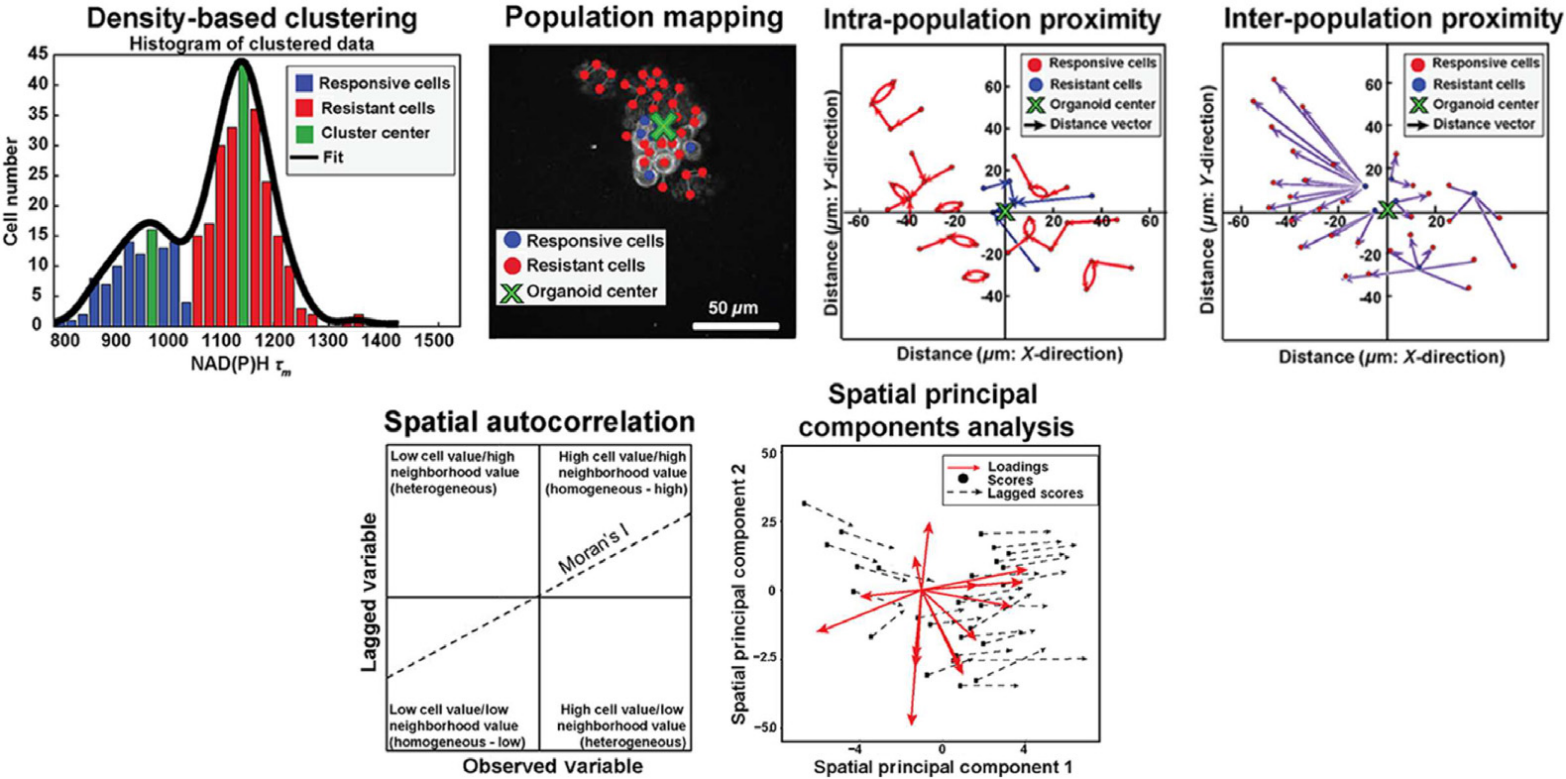
Spatial analysis of fluorescence lifetime distribution. Spatial statistical analyses can quantify spatial heterogeneity in fluorescence lifetimes. Here, spatial heterogeneity in cell-level autofluorescence lifetimes is used as an example. (a) Density-based clustering defines cell subpopulations that are mapped back onto lifetime images. Relative proximity measurements define spatial distributions within (intrapopulation proximity) and between (interpopulation proximity) cell subpopulations. (b) Multivariate spatial heterogeneity is quantified with spatial autocorrelation and spatial principal components analysis. Adapted under CC BY-4.0 with permission from Ref. 234.

Histograms of lifetime values can be plotted across numerous objects for population distribution analysis. This approach visualizes heterogeneity within an object class (e.g., cells or mitochondria) under basal conditions or in response to perturbations. Histograms can be fit with population density models to summarize the distribution of objects and to identify distinct subpopulations of objects. Gaussian mixture modeling is a common population density modeling approach in which multiple Gaussian probability density functions are iteratively fit to each frequency histogram [Fig. 7(a)].^227^^,^^230^ Goodness of fit is assessed by the minimum Akaike information criterion.^230^^,^^231^ However, this approach is limited by assumptions about the number of populations within the data and the Gaussian distribution of the data.^232^^,^^233^ Approaches have been developed to circumvent these assumptions, including density-based clustering [Fig. 8(a)].^235^ Density-based clustering defines subpopulations within data such that the highest density datapoints define the cluster for the nearest remaining points.^235^ Previous studies have shown that population distribution analysis of autofluorescence lifetimes can classify cell types, drug response, and disease states [Fig. 7(b)].^32^^,^^227^^–^^229^^,^^236^^–^^238^ In addition, lifetime distributions can identify objects without prior segmentation. Variations in lifetime distributions have identified other molecular features such as tagged neurons in *C. elegans* and metabolic activities within tumors.^239^^,^^240^ Overall, population distribution analysis provides unique insights into sample heterogeneity.

Heterogeneity in lifetime measurements is commonly quantified from coefficients of variation (CV).^179^^,^^241^^–^^245^ The CV is the standard deviation divided by the mean of a measurement, which enables comparisons of variability between samples. However, the CV does not define whether distinct subpopulations exist within the lifetime data. Alternatively, quantitative metrics of heterogeneity can be derived from population density models, so the behavior of subpopulations can be compared between conditions. A heterogeneity index (H-index) was previously defined to quantify cell subpopulations of fluorescence lifetimes using population density models of cells *in vivo* in head and neck cancer.^228^ This H-index is based on the Shannon diversity index, widely used in ecological studies, and is defined as

$$H\text{-index}=-\underset{i}{\sum }d_{i}p_{i}\;\ln\;p_{i},$$ (19)

where $d_{i}$ is the distance between the medians of each subpopulation and the median of the overall distribution and $p_{i}$ is the proportion of each subpopulation [Fig. 7(a)]. Here, increases in the H-index reflect increases in the number of subpopulations within a sample, increases in the separation between subpopulations, and equality of population proportions. This H-index continues to be adapted for different applications. Specifically, a weighted heterogeneity index (wH-index) was developed to assess metabolic heterogeneity across *in vitro* and *in vivo* breast cancer models.^229^ The wH-index includes subpopulation standard deviations and is defined as

$$wH\text{-index}=\sum [1-p_{i}\;\ln(p_{i}+1)]\times (\sigma_{i}+d_{i}),$$ (20)

where $\sigma_{i}$ is the standard deviation of each subpopulation and p and d denote their proportions and distances from the overall median of the distribution, respectively [Fig. 7(a)].

These heterogeneity metrics have provided valuable insight into diversity within biological systems. The H-index of cell autofluorescence *in vivo* in tumors shows more homogeneous activity across cells (lower H-index) for treatments that significantly reduce tumor volume (combination treatment), and more heterogeneous activity across cells (higher H-index) for treatments that do not change tumor volume (cetuximab or cisplatin alone) [Fig. 7(c)]. In addition, wH-index values of cell autofluorescence for control and treated conditions are similar between *in vitro* tumor organoids and *in vivo* tumors, indicating similar treatment-induced changes in metabolic heterogeneity *in vivo* and *in vitro* for the same tumor model^229^ [Fig. 7(d)]. Collectively, these studies show that quantitative metrics of fluorescence lifetime heterogeneity provide powerful tools to study diversity in biological systems.

### 3.3 Analysis of the Spatial Distributions of Fluorescence Lifetimes

False-colored fluorescence lifetime images can be generated from curve fitting parameters (e.g., $\tau_{1}$, $\tau_{2}$, $\alpha_{1}$, and $\alpha_{2}$) or phasor values (e.g., g and s).^175^^,^^205^ These images are used for qualitative assessments of molecular distribution in biological samples. For example, FLIM images can map lifetime sensors of intracellular molecules (e.g., magnesium, calcium, chromatin, myoglobin, and antigens), pH, oxygen, or temperature.^246^^–^^248^ FLIM images of endogenous fluorophores provide qualitative information on the distribution of subcellular and cellular metabolism, biogenesis, and structure.^51^^,^^80^^,^^205^^,^^209^^,^^243^^,^^249^^–^^252^ Furthermore, tissue-level lifetime images can distinguish cellular compartments across diverse tissue types (e.g., stroma, endothelium, epithelium, and cancerous tissue).^11^^,^^208^^,^^241^^,^^253^^,^^254^

Quantitative metrics of spatial heterogeneity have also been developed for FLIM. Spatial statistical analyses have quantified cell-level spatial heterogeneity in autofluorescence lifetimes across *in vitro* and *in vivo* tumor models. This approach uses density-based clustering to identify populations with distinct lifetimes, map them back to image space, and then extract proximity measurements to assess spatial distributions within a population and between populations [Fig. 8(a)].^234^^,^^235^ Multivariate spatial autocorrelation and spatial principal components analysis can further define patterns based on multiple fluorescence lifetime fit parameters and multiple fluorophores [e.g., NAD(P)H and FAD] [Fig. 8(b)]. Additional quantitative methods have been developed to evaluate spatial variations in intracellular fluorescence, though these have not been translated for lifetime data. For example, QuantEv measures the localization of fluorescently tagged proteins as a function of the global structure of a cell,^255^ and a similar approach was designed for spatial analysis of GFP-expressing plant Golgi proteins.^256^ Quantitative methods to assess fluorescence lifetime spatial distributions will be critical to exploiting the wealth of information in FLIM images.

### 3.4 Multiparametric Analysis of Fluorescence Lifetime Data

Fluorescence lifetime images usually have multiple variables per pixel (e.g., curve fit parameters, fluorescence intensity, and phasor values) that can be used in multivariate classification models to identify distinct cell subsets or functions. For example, partial least squares–discriminant analysis of autofluorescence lifetimes has been used to classify cell-cycle state in heterogeneous samples.^257^ Specifically, this model included NAD(P)H and FAD fluorescence lifetimes and intensities to separate apoptotic, proliferating, and quiescent tumor cells in FLIM images. Other studies used discriminant analysis of fluorescence intensity, lifetime, and morphological parameters to classify cell types (keratinocytes, adipocytes, myoblasts, cardiomyocytes, and stem cells) in response to metabolic perturbations (growth factor and nutrient starvation/supplementation and environmental stimuli).^258^ Multivariate FLIM analysis can also use more complex models including nonlinear classifiers (e.g., logistic regression and random forests) and CNNs. These models also achieve high accuracy for multigroup classification based on autofluorescence lifetimes, specifically for T-cell subtypes and activation states (e.g., quiescent/activated, CD3/CD4/CD8 coexpression).^259^^,^^260^ These studies illustrate the strength of multivariate classification models based on fluorescence lifetime data, which provide robust separation of cell types and cell function.

## 4 Examples of FLIM in Biology and Medicine

Numerous studies have used FLIM to understand molecular features of biological systems and changes due to disease progression or drug treatment. Below are a few examples of autofluorescence FLIM, FLIM of exogenous molecular probes, and FLIM-FRET.

### 4.1 Autofluorescence FLIM Applications

#### 4.1.1 *In vivo* autofluorescence FLIM

Numerous sources of molecular contrast make FLIM attractive for *in vivo* imaging. One of the earliest *in vivo* FLIM studies was performed with intrinsic sources of contrast in human skin. Koenig et al. investigated changes in autofluorescence and SHG that occur with human skin disease *in vivo*.^33^ SHG is a frequency doubling process with instantaneous lifetime (for a review of SHG, see Ref. 261). Collagen fibers create strong SHG signals in skin. Koenig et al. showed that FLIM resolves changes in the autofluorescence lifetime of skin cells within different layers of the skin. Furthermore, FLIM detected cells that had become diseased due to melanoma or fungal infections.^33^ This study pioneered the use of FLIM for clinical applications.

**Fig. 9:**
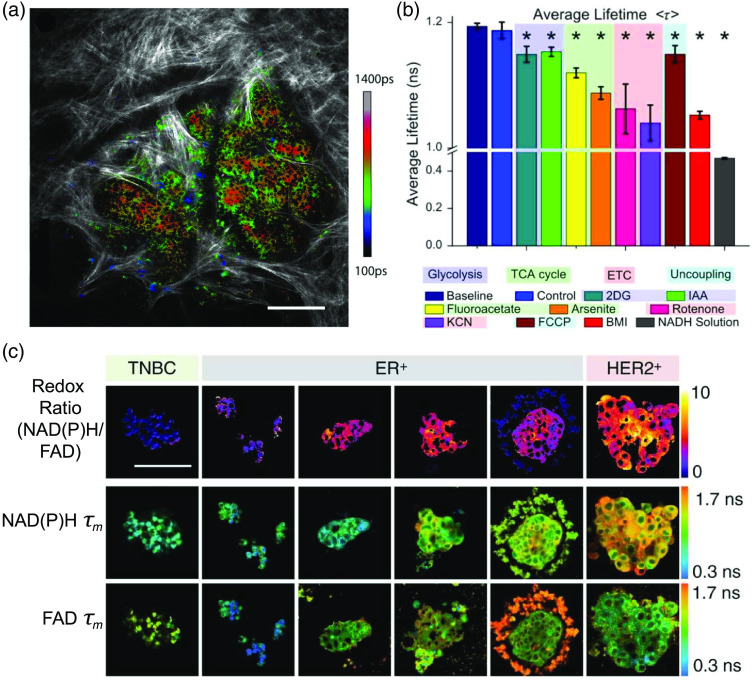
Autofluorescence FLIM applications. (a) NAD(P)H FLIM of a mammary mouse tumor (heatmap) overlaid on an SHG image of collagen (grayscale). Scale bar=100  μm. Adapted with permission from Ref. 264 (b) Mean NAD(P)H lifetimes in solution and in the rat cortex *in vivo* after metabolic inhibition. [2DG, 2-deoxy-d-glucose; IAA, iodoacetic acid; KCNm, potassium cyanide; FCCP, carbonyl cyanide-4-(trifluoromethoxy)phenylhydrazone; BMI, bicuculline methiodide; ETC, electron transport chain.] * indicates significantly different from baseline *in vivo* measurement; Error bars indicate standard error across all pixels over all measurements. Reproduced with permission from Ref. 7 (c) Optical redox ratio [NAD(P)H/FAD; first row], NAD(P)H $\tau_{m}$ (second row), and FAD $\tau_{m}$ (third row) images of organoids generated from primary human breast tumors obtained from resection surgeries. TNBC, triple negative breast cancer; ER, estrogen receptor. Scale bar=100  μm. Adapted with permission from Ref. 265.

Similarly, the first *in vivo* FLIM studies in animal models also focused on autofluorescence. Skala et al. identified differences in the autofluorescence lifetime between normal, low-grade, and high-grade precancerous epithelia in the hamster cheek pouch *in vivo*.^241^^,^^262^ Later, *in vivo* FLIM was used to predict treatment response in mouse tumor models. Specifically, NAD(P)H lifetime changes were found to directly correlate to standard tumor response measurements (i.e., tumor volume).^228^ Importantly, FLIM detected treatment-induced changes in tumors *in vivo* only 2 days post-treatment, which is earlier than detectable changes in tumor volume [6 days post-treatment, Fig. 7(c)]. A recent study also demonstrated that *in vivo* FLIM can measure the efficacy of chemotherapy agents in a mouse model of colorectal cancer.^263^ Furthermore, autofluorescence FLIM can capture metabolic features of specific cell types *in vivo*. Work by Szulczewski et al. indicated that macrophages have a fluorescence lifetime that differs from mammary tumor cells such that macrophages can be identified and monitored *in vivo* without labels^264^ [Fig. 9(a)]. Other *in vivo* applications of autofluorescence FLIM focus on metabolism in the mouse brain. For example, NAD(P)H lifetimes reveal metabolic preferences in the brain using a well-defined set of inhibitors that target-specific metabolic reactions^7^^,^^266^ [Fig. 9(b)].

In addition, autofluorescence FLIM has been performed in numerous non-mammalian *in vivo* models to study organs and whole-body processes that are not easily visualized in mammals. For example, the metabolic gradient along the germline of *C. elegans* was visualized with autofluorescence FLIM,^6^ which provided new insights into metabolic changes with germline differentiation. FLIM has also been performed in plants such as *Arabidopsis*, where FLIM estimated vacuolar pH inside intact plant cells with the lifetime of anthocyanin.^267^

#### 4.1.2 Three-dimensional *in vitro* autofluorescence FLIM

3-D *in vitro* cultures, including organoids and cell constructs within microdevices, have also been assessed with FLIM. Optical sectioning techniques such as CLSM and MP-LSM are especially attractive for FLIM of 3-D cultures due to their high spatial resolution and volumetric imaging capabilities. Numerous cancer studies have focused on predicting *in vivo* drug response using primary tumor organoids. These organoids retain all of the cells of the original tumor in a 3-D matrix so that *in vivo* cell–cell interactions and relevant gradients of oxygen, nutrients, and drugs are preserved.^268^^–^^271^ For example, MP-LSM FLIM indicates that autofluorescence lifetimes in primary tumor organoids can predict *in vivo* response in mouse models across a range of treatment conditions in breast^265^ and head and neck cancer.^272^ Furthermore, FLIM can detect differences in the metabolism of primary patient-derived tumor organoids based upon their surface marker expression [Fig. 9(c)]. FLIM has been used to investigate treatment response in patient-derived tumor organoids across multiple cancer types including breast,^265^ pancreatic,^273^ and colorectal cancer.^274^ In addition, FLIM of colorectal cancer organoids was used to inform a patient treatment regimen.^274^ Organoids provide important 3-D architecture for *in vitro* studies, but microdevices improve the relevance of 3-D cultures by mimicking *in vivo* structures. Specifically, FLIM monitored changes in the metabolism of ductal carcinoma *in situ* cells during invasion in a lumen microdevice model. FLIM captured changes in metabolism based on the position of a cell within the lumen or invading branch.^275^

Tissues *ex vivo* have also been imaged to determine whether FLIM can guide surgical resection of tumors.^276^ First, Lukina et al. compared NAD(P)H FLIM of *in vivo* and *ex vivo* samples using mouse models of colorectal cancer, lung carcinoma, and melanoma to determine optimal tissue maintenance protocols to preserve *in vivo* signals within *ex vivo* samples. Then, Lukina et al. used these protocols to perform NAD(P)H FLIM in postoperative samples obtained from colorectal cancer patients and found significant differences in NAD(P)H lifetimes between normal and malignant specimens.

#### 4.1.3 Autofluorescence FLIM in two-dimensional samples

Autofluorescence FLIM of 2-D *in vitro* cultures provides simple and repeatable systems to test perturbations of autofluorescence lifetime properties. For example, Walsh et al. showed that NAD(P)H lifetimes can detect metabolic differences due to breast cancer subtype.^236^ In addition, the fluorescence lifetime of NAD(P)H correlates with the differentiation potential of neural progenitor and stem cells.^190^ Similarly, changes in the relative fluorescence lifetimes of NAD(P)H and lipid droplet associated granules discriminated differentiated and undifferentiated human embryonic stem cells,^277^ as well as human induced pluripotent stem cell-derived cardiomyocytes under oxidative stress.^278^ Further autofluorescence FLIM studies in 2-D culture discriminated activation states in multiple types of immune cells including macrophages^209^ and T cells.^259^

Finally, autofluorescence FLIM can resolve subcellular features to study intracellular dynamics, including communication between organelles, subcellular features of whole cell processes such as cell division, and bioenergetic demands of different cell types. Mitochondrial organization is often altered to accommodate cellular bioenergetics and biosynthetic demands. Changes in metabolism are also a hallmark of many diseases including cancer. Therefore, mitochondrial imaging has been especially popular for subcellular FLIM applications. Fluorescent dyes such as TMRE can measure mitochondrial membrane potential, which is closely related to cell health.^279^ However, mitochondrial dyes can alter cellular respiration,^280^ and therefore label-free methods are in development. NAD(P)H and FAD fluorescence signals are brightest in the mitochondria, which enables label-free visualization of mitochondria. Pouli et al. showed that FLIM of NAD(P)H and FAD can capture rapid changes in mitochondrial spatial dynamics and metabolism using high-resolution imaging of individual mitochondria within cells.^281^

### 4.2 FLIM of Exogenous Molecular Probes

#### 4.2.1 Exogenous molecular probes for *in vivo* applications

Numerous optical probes have been developed for both *in vivo* and *in vitro* applications to capitalize on the sensitivity of FLIM to physical conditions, including viscosity,^282^ temperature,^283^ acidity,^284^ and oxygenation.^104^^,^^247^^,^^285^ Additional molecular probes have been generated that allow for FLIM-based monitoring of drug delivery.

**Fig. 10:**
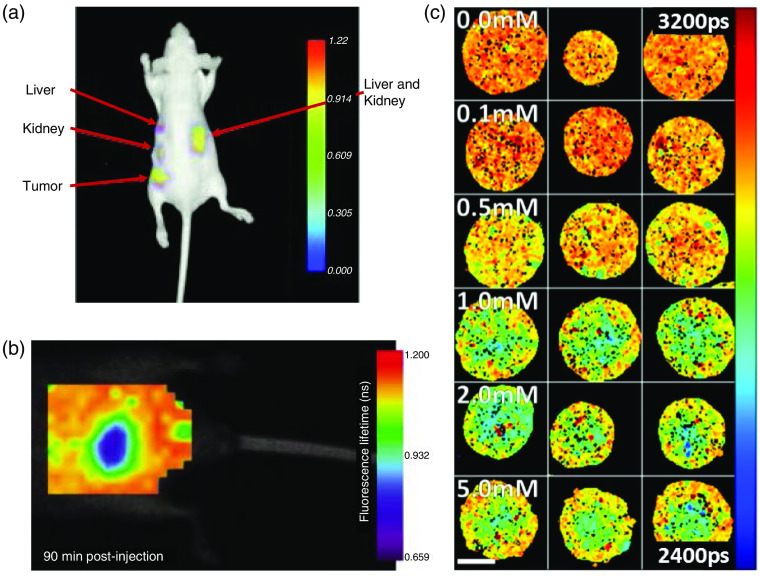
Molecular probes for FLIM and FLIM-FRET. (a) Near-infrared fluorescence lifetime image of Cyp-GRD distribution (heatmap) in an A549-tumor-bearing mouse at 24-h postinjection. Adapted with permission from Ref. 287 (b) Fluorescence lifetime (heatmap) of mouse abdomen acquired 90 min after intravenous injection of LS-288. The low fluorescence lifetime region in the center of the abdomen is the filled urinary bladder. Adapted with permission from Ref. 288. (c) FLIM maps of the weighted mean fluorescence lifetime of T2AMPKAR-NES, a sensor for AMPK activation, in HEK293 spheroids. The blue end of the colormap indicates increased AMPK activation. Scale bar=100  μm. Adapted with permission from Ref. 289.

Mouse models are widely used for *in vivo* FLIM studies of exogenous molecular probes. Ardeshirpour et al. detected mouse tumors *in vivo* that express human epidermal growth factor receptor (HER2) with FLIM of a fluorescent anti-HER2 antibody.^286^ Similarly, FLIM showed that the near-infrared fluorescence dye cypate localizes to mouse tumors *in vivo* [Fig. 10(a)]. FLIM of two fluorophores, cypate and bacteriochlorophyll, can identify the unique distribution of each fluorophore *in vivo*,^239^^,^^287^^,^^290^ which is difficult with intensity-based imaging alone. FLIM has also evaluated renal function in mice using the fluorescence lifetime reporter LS-288, which has a distinct lifetime when free in solution vs. bound to proteins. This approach provides contrast between the protein-rich viscera and the mostly protein-free bladder in mice *in vivo* [Fig. 10(b)].^288^ Furthermore, pH-sensitive fluorescence lifetime probes that provide a nonterminal method to quickly determine the acidity of a region *in vivo* have been developed.^291^ Overall, FLIM in conjunction with the development of these sophisticated probes is promising in cancer detection and other *in vivo* applications.

In non-mammalian *in vivo* models, fluorescence lifetime probes that change with both temperature^292^^,^^293^ and concentration of ions have been developed. For example, Zhang et al. generated a phosphorescent lifetime probe that is temperature dependent and demonstrated this temperature dependence *in vivo* in a zebrafish model.^283^ Another example of a non-mammalian application of fluorescence lifetime probes includes imaging chloride ion concentrations in cockroach salivary glands done by Hille et al.^294^

#### 4.2.2 *In vitro* molecular probe FLIM

Many fluorescence lifetime probes exist for *in vitro* applications to measure whole cell changes and localize molecular trafficking within a cell. For example, a fluorescence lifetime probe was developed to track the location and use of ${\mathrm{Zn}}^{2+}$ within a cell. These probes can be localized to understand ion use within individual organelles. Other fluorescence lifetime probes have been developed to detect intracellular prodrug trafficking,^295^ as well as pH^296^ and oxygenation changes. Oxygen sensing via phosphorescent lifetime imaging has become a well-established method to monitor intracellular oxygen tension. Furthermore, simultaneous measurement of NAD(P)H FLIM and oxygen sensing by phosphorescence lifetime imaging of Ruthenium tris-(2,2′-bipyridyl) has also been demonstrated in 2-D cell cultures.^297^

### 4.3 FLIM-FRET Applications

#### 4.3.1 FLIM-FRET for *in vivo* applications

Finally, FLIM can be used to better capture extracellular and subcellular interactions on the nanoscale both *in vivo* and *in vitro* via FLIM-FRET. FLIM-FRET interactions can be used to measure protein activity, gene regulation, and subcellular dynamics. For example, an activatable FRET probe has been developed with a donor–acceptor pair that can be cleaved by matrix metalloproteinases (MMP). This probe was used in a mouse model of breast cancer to monitor MMP activity.^298^ FLIM-FRET has also identified patterns in RhoA activity *in vivo* using a GFP-RFP Raichu-RhoA reporter. These studies found that active RhoA, which is associated with cellular cytoskeleton organization, has subcellular localization to the leading edge of invasion in a pancreatic cancer mouse model.^299^

FLIM-FRET probes have also been used in non-mammalian *in vivo* models including plants and zebrafish. In *Arabidopsis* roots, FLIM-FRET probes have been developed to investigate the role of transcription factors that regulate plant cell fates.^300^ In zebrafish, FLIM imaged a time-course of apoptosis after radiation treatment in 3-D over the entire zebrafish body using a FRET sensor,^301^ which provided an important whole-body context for the apoptotic process. These are just a few of the many non-mammalian *in vivo* applications of FLIM-FRET.

#### 4.3.2 FLIM-FRET for *in vitro* applications

Subcellular dynamics can also be monitored with FLIM-FRET *in vitro*. T2AMPKAR-NES is a FLIM-FRET sensor for AMPK activation that was shown to detect spatial changes in the activity of AMPK between the inner and outer layers of human embryonic kidney spheroids in a 3-D culture [Fig. 10(c)].^289^ FLIM-FRET has also been used in a 2-D culture of mouse pituitary cells to detect dimerization between the transcription factor CAATT and the enhancer binding protein alpha. This dimerization corresponds with increased gene expression and adipogenesis.^302^ Finally, autofluorescence FLIM-FRET can detect molecular interactions within live cells as well, specifically between NAD(P)H and tryptophan. In these studies, tryptophan is the FRET donor and NAD(P)H is the FRET acceptor [i.e., NAD(P)H quenches tryptophan fluorescence]. These studies found that doxorubicin increases the abundance of FRET interactions between tryptophan and NAD(P)H.^303^ New developments in super-resolution FLIM can localize molecular features within smaller structures and is growing in popularity along with other super-resolution techniques.^133^^,^^304^

### 4.4 Challenges and Solutions in FLIM Applications

FLIM provides insight into molecular features of living systems, yet challenges remain. FLIM instrumentation is more costly than intensity-based measurements. In addition, FLIM analysis requires specific expertise, and the computational cost of FLIM analysis is often higher than intensity-based imaging. Furthermore, FLIM acquisition is generally slower than intensity imaging because more photons are needed to accurately estimate a fluorescence lifetime for each pixel. This can be limiting for biological processes that occur rapidly.^305^ Additionally, fluorescence lifetimes are affected by numerous factors (e.g., molecular interactions/binding activity, environmental factors such as pH, temperature, and viscosity), which can complicate the interpretation of fluorescence lifetime changes in biological systems. Like all light microscopy, FLIM suffers from scattering that limits SNR and resolution at deep imaging depths and/or highly scattering samples. The effects of scattering can be improved with MP-LSM, but imaging depths are still limited to <2  mm in most tissues.^106^ For *in vivo* applications, motion artifacts from animal breathing and heartbeat require specific sample preparation and/or image gating to maintain quality during FLIM acquisition,^306^ also in a similar fashion to other light microscopy techniques.

Endogenous fluorophores have quantum yields that are orders of magnitude lower than traditional dyes,^76^ which presents challenges for autofluorescence FLIM. Furthermore, disentangling the contributions from multiple endogenous fluorophores can be difficult when lifetime values overlap, such as NADH and NADPH or FAD and FMN.^49^^,^^307^^–^^310^ Numerous drugs also naturally fluoresce, and these properties must be known when measuring fluorescence lifetime changes due to drug treatment. For example, Mohammed et al. could not separate the overlapping lifetimes of NAD(P)H and zinc-oxide nanoparticles, so their contribution was combined into one lifetime component.^311^ Other experimental parameters including cell density, media conditions, 2-D versus 3-D culture, and coatings on culture dishes can have significant effects on autofluorescence lifetime values.^51^^,^^205^^,^^237^ To minimize these challenges, care should be taken to keep parameters consistent across samples and a daily control sample should be imaged. Furthermore, drugs that can directly modulate autofluorescent molecules, such as duroquinone, which has been shown to modulate the ratio of NAD+ to NADH, might aid in interpretation.^312^

FLIM of molecular probes is often challenged by the nonspecific binding of the probe. Molecular probes *in vivo* also suffer from high background due to autofluorescence. FLIM probes with long lifetimes (>8  ns) can remove autofluorescence background, but acquisition must be optimized for these longer lifetimes. FLIM-FRET probes must also be designed to accurately measure protein interactions without interfering with the interaction, which is especially difficult when the labeled molecules are overexpressed and likely disrupt the normal state of the cells.^313^ Therefore, careful management of the experimental conditions and appropriate controls are needed for robust FLIM studies. Overall, FLIM is an enabling technique that requires specific training for reproducible results and appropriate data interpretation.

## 5 Conclusion

FLIM is a widely used tool for biomedical imaging and has advanced the field of microscopy in the past few decades. In this review, we discussed FLIM as a technique to measure biophysical changes at the molecular scale. FLIM coupled with fluorescence lifetime probes can quantify chemical and physical changes to molecules including changes in temperature, viscosity, pH, and others. Unlike intensity-based measurements, lifetime is self-referenced and independent of the absolute number of photons. Therefore, FLIM is not corrupted by variations in intensity between pixels.

FLIM instrumentation can be deployed in either the time-domain or frequency-domain, which generates either a photon timing histogram or a phase-frequency plot to measure the exponential decay rate of the fluorescence. Analytical equations describe the decay rate and fluorescence lifetime. FLIM images can be acquired either in a WFI scheme using a camera-based detection or by raster-scanning a focused point of excitation across a sample using a single channel detector. Instrumentation schemes are flexible and can be optimized for the desired field of view, spatial–temporal resolution, imaging speed, and other considerations. Multiphoton FLIM provides a unique tool for 3-D optical sectioning and deeper imaging depth into tissues, which is especially advantageous for *in vivo* and *in situ* imaging. Current fast-FLIM systems use electronics with short dead times to increase frame rates for medical applications in surgery. Algorithms for FLIM analysis are under rapid development to improve image segmentation, quantify multidimensional heterogeneity, and perform multiparametric analysis. These computational tools unravel spatial and molecular features of cellular physiology that are not apparent with qualitative observation of FLIM images.

Numerous biomedical applications were discussed including autofluorescence FLIM as a label-free method to monitor metabolism and protein–enzyme interactions with the endogenous fluorophores NAD(P)H and FAD. Autofluorescence FLIM has provided insight into metabolism in cancer, stem cells, immune cells, and the brain across diverse sample types including 3-D organoids, microfluidic physiological systems, *in vivo* mouse models, and human skin. FLIM-FRET sensors have also quantified molecular interactions related to cellular signaling, cellular proliferation, and cytokinesis. In the future, FLIM technologies, analysis, and applications will continue to develop toward advancements in biological research and clinical assessments.
